# Spindle pole cohesion requires glycosylation-mediated localization of NuMA

**DOI:** 10.1038/s41598-017-01614-6

**Published:** 2017-05-03

**Authors:** Jérémy Magescas, Lucie Sengmanivong, Amandine Viau, Adeline Mayeux, Tien Dang, Martine Burtin, Ulf J. Nilsson, Hakon Leffler, Françoise Poirier, Fabiola Terzi, Delphine Delacour

**Affiliations:** 1 Cell Adhesion and Mechanics, Institut Jacques Monod (IJM), CNRS-UMR7592, Paris Diderot University, 15 Rue Hélène Brion, 75013 Paris, France; 2 Institut Curie - Centre de Recherche, PSL Research University, Membrane Dynamics and Mechanics of Intracellular Signaling laboratory, Paris, France; 3 INSERM U1151 - CNRS UMR 8253, Université Paris Descartes, Institut Necker Enfants Malades, Département Croissance et Signalisation, Hôpital Necker Enfants Malades, 149 Rue de Sèvres, 75015 Paris, France; 4 Galectin chemistry and biology, Centre for Analysis and Synthesis, Department of Chemistry, Lund University, POB124, SE-22100 Lund, Sweden; 5 Section MIG Microbiology, Immunology, Glycobiology, Department of Laboratory Medicine, Faculty of Medicine, Lund University, POB124, SE-22100 Lund, Sweden; 6 Morphogenesis, homeostasis and pathologies, Institut Jacques Monod (IJM), CNRS-UMR7592, Paris Diderot University, 15 Rue Hélène Brion, 75013 Paris, France

## Abstract

Glycosylation is critical for the regulation of several cellular processes. One glycosylation pathway, the unusual *O*-linked β-*N*-acetylglucosamine glycosylation (*O*-GlcNAcylation) has been shown to be required for proper mitosis, likely through a subset of proteins that are *O*-GlcNAcylated during metaphase. As lectins bind glycosylated proteins, we asked if specific lectins interact with mitotic *O*-GlcNAcylated proteins during metaphase to ensure correct cell division. Galectin-3, a small soluble lectin of the Galectin family, is an excellent candidate, as it has been previously described as a transient centrosomal component in interphase and mitotic epithelial cells. In addition, it has recently been shown to associate with basal bodies in motile cilia, where it stabilizes the microtubule-organizing center (MTOC). Using an experimental mouse model of chronic kidney disease and human epithelial cell lines, we investigate the role of Galectin-3 in dividing epithelial cells. Here we find that Galectin-3 is essential for metaphase where it associates with NuMA in an *O*-GlcNAcylation-dependent manner. We provide evidence that the NuMA-Galectin-3 interaction is important for mitotic spindle cohesion and for stable NuMA localization to the spindle pole, thus revealing that Galectin-3 is a novel contributor to epithelial mitotic progress.

## Introduction

Glycosylation encompasses a broad and varied range of post-translational modifications (PTMs)^1^. While other PTMs such as phosphorylation are simple, glycosylation PTMS have more complex structures with a highly variable composition and arrangement, and it is thus difficult to infer a single function for a specific glycosylation. While most glycosylations occur in the Golgi apparatus, an unusual type of glycosylation, called *O*-linked β-*N*-acetylglucosamine glycosylation (*O*-GlcNAcylation), occurs in the cytosol in a ubiquitous and reversible manner, similar to phosphorylation^2^. Two proteins control *O*-GlcNacylation: the *O*-GlcNAmine transferase, to add the *O*-GlcNAc moiety, and the *O*-GlcNAminase, to remove it. It is not yet completely understood how these proteins are regulated, nor how they target or ignore glycosylation sites in cytosol substrates^3, 4^. A large panel of proteins are *O*-GlcNAcylated, and the PTM is dynamic, changing in response to glucose intake or different signalling pathways^4, 5^. One current hypothesis is that this PTM would act as a tuning mechanism to regulate signalling pathway activity, in part by competing with phosphorylation, for their shared threonine/serine modification sites^5, 6^. However, recent studies have suggested that *O*-GlcNAcylation serves an active role beyond blocking phosphorylation. In addition, *O*-GlcNAcylation has been reported to be required for proper mitosis^7–9^, and only a specific subset of substrate proteins are *O*-GlcNAcylated during metaphase^8^. This finding indicates clearly that glycosylation contributes to the regulation or progression of cell division process, and suggest that the link between cancer establishment/progression and glycosylation may occur through direct action on mitosis via modification of M-phase proteins.

Lectins are glycosylation “readers”, but, so far, no lectin has been described to recognize *O*-GlcNAcylated motifs. A small number of lectins are found soluble in the cytosol, and the Galectin family is among them. Over the years, Galectins have been implicated in a wide range of implications in both extra- and intracellular biologic processes^10^. The Galectin family is highly conserved and is comprises 15 members, initially described based on their affinity for ß-galactosides. They are small (from 14 to 36 kDa), non-glycosylated, and soluble, and they are found inside and outside of cells. Three different structural configurations can be distinguished: the prototypic Galectins (Galectin-1, -2, -5, -7, -10, -11, -13, -14, -15), which are exclusively composed of a pair of unique Galectin carbohydrate-recognition domain (CRD); the tandem-repeat Galectins (Galectins-4, -6, -8, -9, -12), which have two CRDs linked through a short linker peptide, and the chimeric Galectin (Galectin-3), with one CRD domain and a unique N-terminal tail. Galectin-3 is highly expressed in epithelial cells and is involved in many processes, including intracellular trafficking and cell cycle signaling^11, 12^. It has been notably described as a transient centrosomal component in interphase and mitotic epithelial cells^13, 14^. In addition, it has recently been shown to associate with basal bodies in motile cilia, where it tightly associates with and stabilizes the microtubule-organizing center (MTOC)^15^. Importantly, perturbation of Galectin-3 expression has been associated with epithelial cancer progression^16, 17^. The correlation between Galectin-3 and epithelial cancer, the localization of Galectin-3 at the MTOC and the apparent importance of glycosylation in cell division process, prompted us to hypothesize that Galectin-3 is required for proper mitosis in a glycosylation-dependent manner.

Here we show that the absence of Galectin-3 leads to severe defects in epithelial tissue architecture, at least in part due to cell division failure. Further investigation highlighted the association of Galectin-3 with the mitotic spindle pole, a robust structure that ensures proper sister chromatide alignment^18–20^. We also find that Galectin-3 interacts with a key mitotic regulator, NuMA (Nuclear Mitotic Apparatus protein), which is required for the establishment and the cohesion of the spindle pole^21^. We report that the Galectin-3/NuMA interaction is functionally important for the spindle pole organization. Altogether our results provide new mechanistic insight into the role of glycosylation in controlling epithelial cell division.

## Results

### Galectin-3 is required for normal tissue organization in mouse kidney after nephron reduction

**Figure 1:**
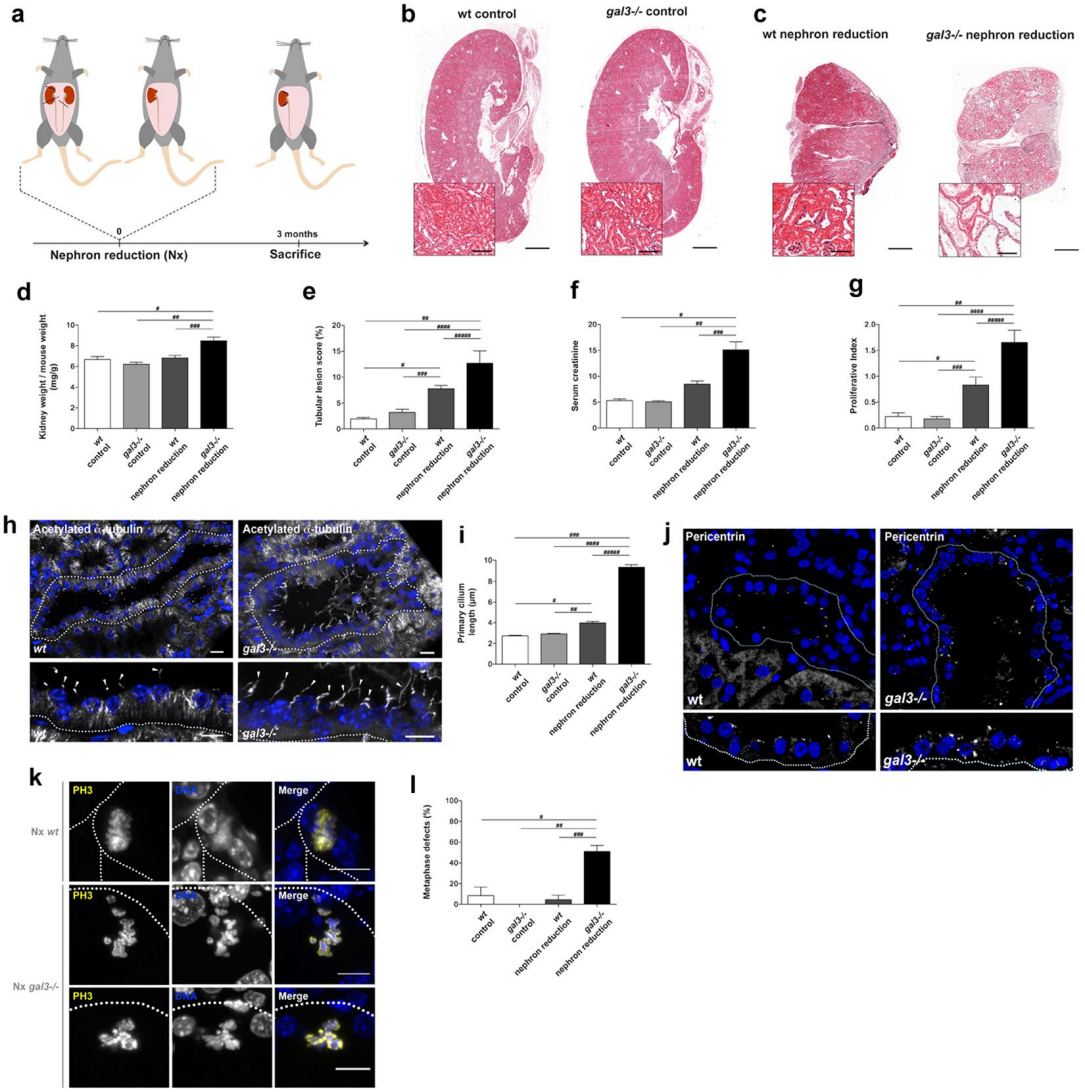
Galectin-3 loss leads to severe tissue disorganization during renal recovery. (**a**) Scheme showing the nephronic reduction procedure. (**b–c**) Histological analyses of sham-operated (*Control*) (**b**) and Nx (**c**) wt and *gal3*−/− kidneys 3 months after surgery. Scale bars, 1 mm; inserts, 100 µm. (**d**) Statistical analysis of the ratio kidney weight over mouse weight in control and Nx, wt and *gal3*−/− kidneys. (**e**) Statistical analysis of tubular dilatations. (**f**) Statistical analysis of the amount of serum creatinine in control and Nx wt and *gal3*−/− kidneys. (**g**) Statistical analysis of the proliferative index in control and Nx wt and *gal3*−/− kidneys. (**h**) Confocal microscopy analysis and 3D rendering of primary cilia (*white arrow arrowhead*) immunostained with monoclonal anti-acetylated α-tubulin antibody (*white*) in Nx wt and Nx *gal3*−/− kidneys. Scale bars, 10 µm. (**i)** Statistical analysis of the primary cilium length in control and Nx kidneys in wt and *gal3*−/− mice. (**j**) Confocal microscopy analysis of the distribution of pericentrin (*white*) in Nx wt and Nx *gal3*−/− kidneys. *N*(_Nx wt_) = 6 mice, *N*(_Nx *gal3*_−/−) = 6. (**k**) Confocal microscopy analysis of mitoses with PH3 immunostaining (*yellow*) in Nx kidneys. *Arrowheads*, irregular metaphasic plate; *arrows*, lagging chromosomes. (**l**) Statistical analysis of the percentage of cells with unconventional metaphasic plate morphology. In the Fig. 1, data are means ± SEM. Renal epithelium was demarcated with a dotted white line. Nuclei were detected by Hoechst 33342 staining (*blue*). Detailed information about quantifications is available in Supplementary Information.

To first test the importance of Galectin-3 in mitosis *in vivo*, we analysed mitotic defects in the mouse kidney, where Galectin-3 has been reported to associate with centrosomes and centrosome-derived organelles^13, 14^. Since the renal epithelium is only renewed at a slow rate, we challenged *gal3*−/− mutant mice with a pathological renal stress to stimulate cell proliferation, using the established experimental model of chronic kidney disease designated “nephron reduction” or “partial nephrectomy” (Nx)^22, 23^. This model, which consists in the excision of 75% of the total renal mass (Fig. 1a), is characterized first by a phase of compensatory growth, then the progressive development of renal lesions, leading to deterioration of the remaining kidney tissue. An increase in epithelial cell proliferation accompanies both the compensatory and the deterioration phase^22, 23^. We previously showed that these events are genetically determined and identified mouse strains that are resistant to renal lesion development, such as the 129/sv background mice used in this study^24, 25^. As expected, remnant kidneys of 129/sv wild-type (Nx wt) mice underwent compensatory growth, but did not displayed renal lesions the 3 months following Nx^24^ (Fig. 1b,c). However, we observed that following Nx, the lack of Galectin-3 (Nx *gal3*−/−) leads to increased kidney weight increase (Fig. 1d) and severe tubular alterations, specifically cyst development, unlike their cyst-free wt littermates (Fig. 1b,c and e). When we assessed renal function by measuring plasma creatinine, we observed a stronger elevation of creatinine concentration occurs in Nx *gal3*−/− mice compared to Nx wt mice, demonstrating renal failure (Fig. 1f). These kind of defects in the morphogenesis and function of renal tubules have also been observed in the context of cystic kidney phenotypes, in both acquired cystic kidney disease (ACKD) and genetic polycystic kidney disease (PKD), where they correlate with defects in cell proliferation, growth of primary cilium and perturbation of mitosis^26–30^. As in these other cystic kidney tissues, we found that Nx *gal3*−/− kidneys exhibit increased cell proliferation (Fig. 1g). The primary cilium growth also overelongates in Nx *gal3*−/− renal interphase cells (Fig. 1h-i), as previously observed in MDCK cyst cultures^14^. In addition, Nx *gal3*−/− cells display numerous pericentrin accumulations in contrast with pericentrin localization in wt cells, suggesting that the absence of Galectin-3 may lead to a centrosomal perturbation with PCM fragmentation or centrosome duplication (Fig. 1j). Interestingly, Nx *gal3*−/− cells show a dramatic increase dysmorphic metaphases plates (i.e. fragmented metaphase plate, triskelion shaped metaphase plate, …). Whereas control metaphase cells display regular metaphase plates, *gal3*−/− metaphase cells harbour ill-shaped metaphase plate in a triskelion-like shape or individual chromosomes escaping the metaphase plate, suggesting the presence of multipolar cells and defective organisation of spindles in Nx *gal3*−/− cells (Fig. 1k-l). These data demonstrate that loss of Galectin-3 leads to severe tissue disorganization during renal recovery and strongly suggest that Galectin-3 promotes robust cell division in sensitized tissue *in vivo*.

### Galectin-3 is required for normal cell division in epithelial cell cultures

**Figure 2:**
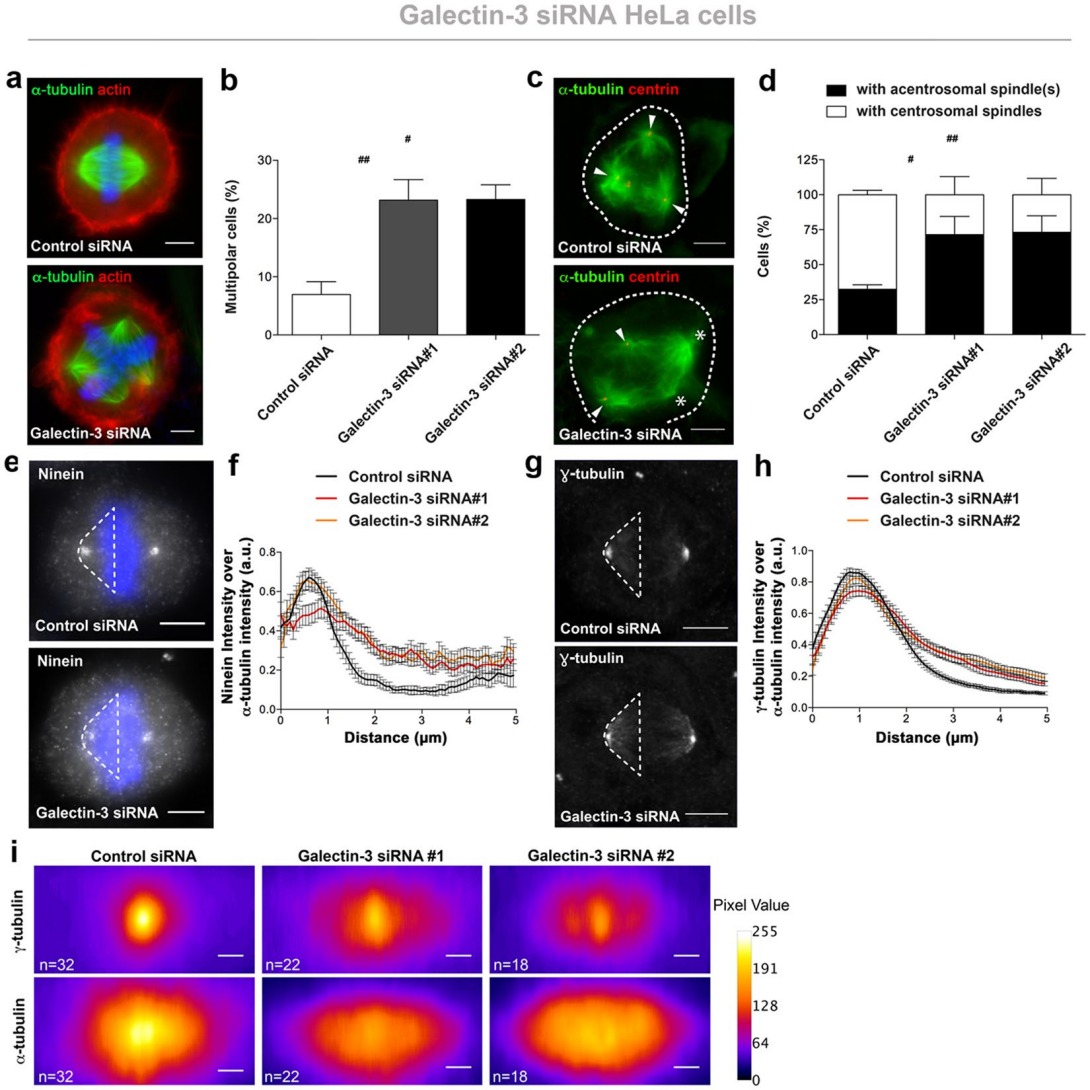
Galectin-3 is required for correct mitoses in epithelial cell cultures. (**a**) Confocal microscopy analysis of mitotic poles after α-tubulin immunostaining and actin detection in Hela cells. Scale bars, 5 µm. (**b**) Statistical analysis of the number of multipolar mitosis. *n*(_Control siRNA_) = 1406 cells, *n*(_Galectin-3 siRNA #1_) = 1291, *n*(_Galectin-3 siRNA #2_) = 1158. Data are means ± SEM. (**c**) Confocal microscopy analysis of centrosomal (*arrowheads*) and acentrosomal (*stars*) mitotic spindles. Cells were immunostained for α-tubulin (*green*) and centrin-3 (*red*). Scale bars, 5 µm. (**d**) Statistical analysis of the number of centrosomal or acentrosomal mitotic spindles. *n* (_Control siRNA_) = 48 cells; *n* (_Galectin-3 siRNA #1_) = 55; *n* (_Galectin-3 siRNA #2_) = 53. Data are means ± SEM. (**e**) Fluorescence microscopy analysis of ninein in control and Galectin-3 siRNA cells. Nuclei were detected by Hoechst 33342 staining (*blue*). A white dotted line specifies the area which has been used for ninein intensity quantification in (**f)**. Scale bars, 5 µm. (**f**) Statistical analysis of the intensity of ninein fluorescence over α-tubulin fluorescence intensity along the mitotic spindle in control (*Control siRNA*, *black line*) and Galectin-3-depleted (*Galectin-3 siRNA#1*, *red line and Galectin-3 siRNA#2, orange line*) metaphase Hela cells. *n* (_Control siRNA_) = 19 cells; *n* (_Galectin-3 siRNA#1_) = 19 cells; *n* (_Galectin-3 siRNA#2_) = 13 cells. (**g**) Fluorescence microscopy analysis of γ-tubulin in control and Galectin-3 siRNA cells. A white dotted line specifies the area which has been used for γ-tubulin intensity quantification in (**h)**. Scale bars, 5 µm. (**h**) Statistical analysis of the intensity of γ-tubulin fluorescence over α-tubulin fluorescence intensity along the mitotic spindle in control (*black line*) and Galectin-3-depleted (*red and orange lines*) metaphase Hela cells. *n*(_Control siRNA_) = 26 cells; *n*(_Galectin-3 siRNA#1_) = 19; *n*(_Galectin-3 siRNA#2_) = 21. (**i)** Fluorescence microscopy analysis of α-tubulin and γ-tubulin (*in pseudocolor spectrum)* at spindle pole in bipolar mitosis. Images are mean projection of xz spindle centered through DNA staining. *n*(_Control siRNA_) = 32 cells; *n*(_Galectin-3 siRNA#1_) = 22; *n*(_Galectin-3 siRNA#2_) = 18. Detailed information about quantifications is available in Supplementary Information.

We next sought to determine if Galectin-3 promotes normal mitosis. Since the *in vivo* system was limiting for this study, we switched to *in vitro* with the use of HeLa cells, a non-ciliated epithelial cell line. We silenced Galectin-3 by transient RNA interference (siRNA; Supplementary Fig. S1a,b). We found that a significant increase in mitosis-related defects occurred in the absence of Galectin-3 as described thereafter^18, 31^. Galectin-3-depleted cells displayed an increased frequency of multinucleated cells (Supplementary Fig. S2a,b) and of supernumerary centrioles at interphase (Supplementary Fig. S3a,b), when compared with controls. Looking more precisely at metaphase cells, multipolar mitoses were more frequent in Galectin-3-depleted cells than in control cells (22.59 ± 2.79% and 22.99 ± 1.97% in both Galectin-3-siRNA cells versus 7.67 ± 1.84% in control cells; Fig. 2a,b). Surprisingly, Galectin-3-silenced cells showed multipolar mitoses with acentrosomal poles more often than control cells (77.59 ± 10.74% and 79.76 ± 4.02% in both Galectin-3-siRNA cells versus 33.92 ± 2.52% in control cells; Fig. 2c,d). These data indicate that the increase in multipolar mitoses in Galectin-3-silenced cells is probably be due to defects in the level of the pericentrosomal matrix (PCM), an improper centrosome clustering or in spindle pole stability rather than due to centrosomal overduplication^18^. To test this, we next analysed the distribution of PCM proteins. We examined ninein and γ-tubulin (Fig. 2e–h), which bind and nucleates microtubules respectively. Ninein appeared less focused at spindle poles and more scattered in the cytoplasm (Fig. 2e,f). γ-tubulin spread along MTs at the level of the spindle pole and spindle in Galectin-3-depleted cells (Fig. 2i,j). We conclude that Galectin-3 is instrumental for normal mitotic spindle formation, and we speculate that it promotes PCM stability at the spindle pole.

### Galectin-3 associates with spindle poles

**Figure 3:**
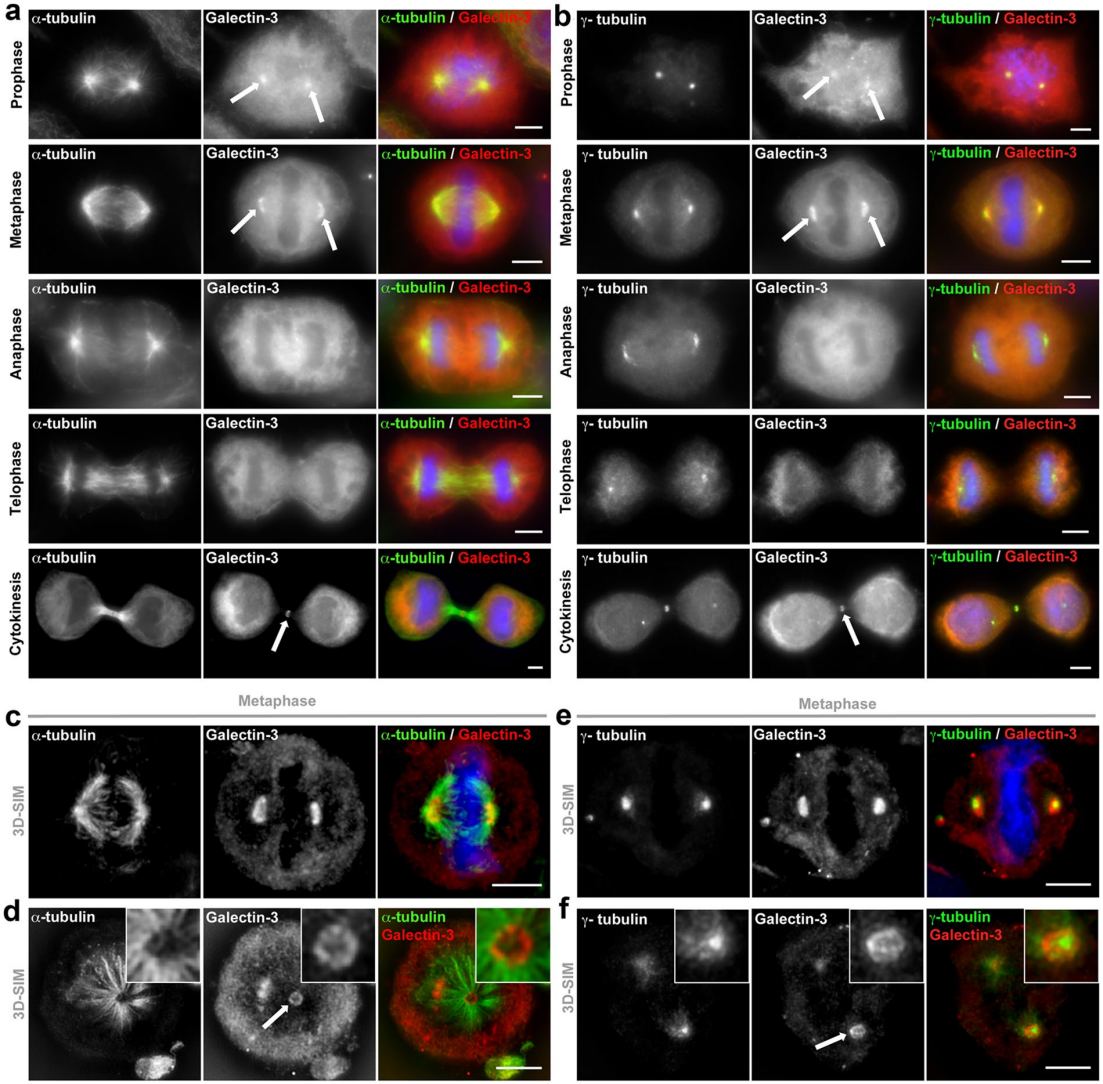
Galectin-3 associates with spindle poles. (**a,b**) Epifluorescence microscopy analysis of Galectin-3 distribution during mitosis. Hela cells were immunostained for Galectin-3 (*red*) and either α-tubulin (**a**) or γ-tubulin (**b**) (*green*). Galectin-3 is located at the spindle poles in prophase and metaphase (*arrows*), and with cleavage furrow in cytokinesis (*arrows*). Nuclei were detected by Hoechst 33342 staining (*blue*). Scale bars, 5 µm. (**c**–**f**) 3D-SIM microscopy analysis of Galectin-3 localization at spindle poles. Hela cells were immunostained for Galectin-3 (*red*) and either α-tubulin (**c,d**) or γ-tubulin (**e,f**) (*green*). Longitudinal (**c** and **e**) and top (**d** and **f**) spindle views are shown and close-ups on spindle pole top views are presented in inserts. Nuclei were detected by Hoechst 33342 staining (*blue*). Galectin-3 is associated with (−) end MTs neighbouring γ-TURC complexes at spindle poles (*arrows*). Scale bars, 5 µm.

To understand how Galectin-3 promotes normal spindle pole formation, we examined its subcellular localization during mitosis (Fig. 3). Although Galectin-3 is primarily cytosolic during mitosis, it is transiently enriched at the spindle pole during prophase and metaphase, and at the cleavage furrow during cytokinesis (Fig. 3a,b, arrows), showing that Galectin-3 exhibits a dynamic distribution, similar to other MTOC-associated proteins during mitosis. For a more detailed understanding of Galectin-3 localization at spindle poles, we used high-resolution microscopy (3D-Structured Illumination Microscopy, 3D-SIM) (Fig. 3c–f) and observed Galectin-3 colocalized with the spindle pole (Fig. 3d, arrow), close to γ-tubulin MT-nucleation complexes (Fig. 3f, arrow). To confirm Galectin-3 association with the PCM, its localization was followed after nocodazole treatment and wash out. Early during metaphase MT regrowth, Galectin-3 localizes close to MT nucleation complexes at the base of MT asters (Supplementary Fig. 4a, arrowheads) and to γ-tubulin foci (Supplementary Fig. 4b, arrowheads). These results show that Galectin-3 closely associates with the spindle MTOC, and we propose that Galectin-3 likely functions at the spindle pole to promote normal spindle formation.

### Galectin-3 and NuMA interact at spindle poles through lectin activity of Galectin-3

**Figure 4:**
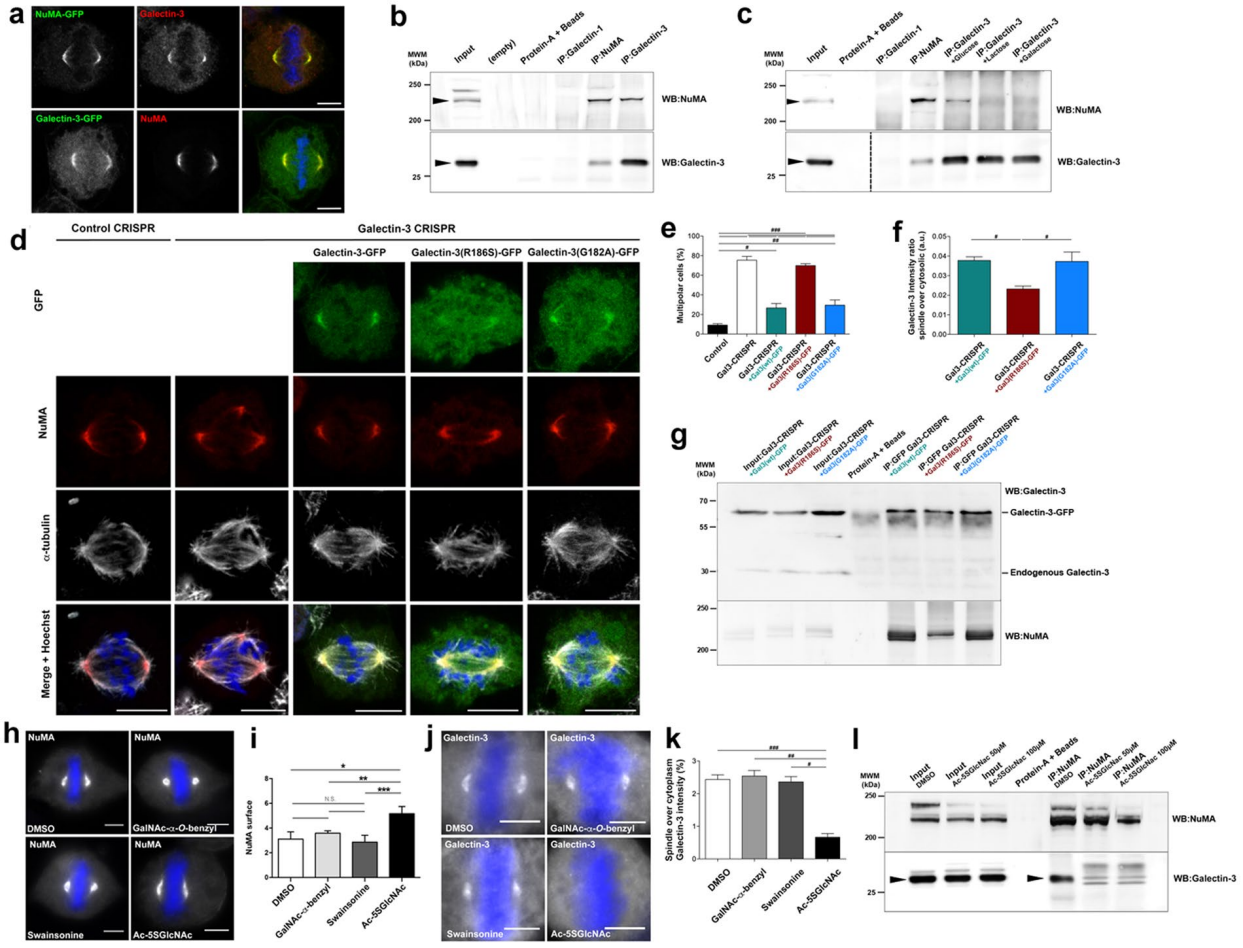
Galectin-3 and NuMA interaction relies on Galectin-3 lectinic activity. (**a**) Confocal microscopy analysis of endogenous Galectin-3 and transfected NuMA-GFP, and *vice versa*. Scale bars, 5 µm. (**b**) Western blot detection of NuMA (*Top blot, black arrowhead*) or Galectin-3 (*Bottom blot, black arrowhead*) after immunoprecipitation from mitotic extracts. Input, 10% of total cell lysate. (**c**) Western blot detection of NuMA (*Top blot, black arrowhead*) and Galectin-3 (*Bottom blot, black arrowhead*) after immunoprecipitation of NuMA or Galectin-3 in presence of Galectin-3-competitive sugars. Input, 10% of total cell lysate. (**d**) Confocal microscopy analysis of NuMA and α-tubulin in control and Galectin-3-depleted cells using CRISPR-Cas9 strategy, after transfection of Galectin-3-GFP, Galectin-3-R186S-GFP or Galectin-3-G182A-GFP. Scale bars, 5 µm. (**e)** Statistical analysis of the number of multipolar cells after transfection of Galectin-3-GFP constructs in control or Galectin-3–depleted cells using CRISPR-Cas9 strategy. (**f**) Statistical analysis of the intensity of Galectin-3-GFP constructs over cytosol. (**g**) Western blot detection of Galectin-3 (*Top blot*) and NuMA (*Bottom blot*) after the immunoprecipitation of GFP from mitotic extracts of Galectin-3-depleted cells using CRISPR-Cas9 strategy, and transfected with Galectin-3-GFP constructs. Input, 10% of total cell lysate. (**h**) Fluorescence microscopy analysis of NuMA distribution in metaphase cells after DMSO or glycosylation inhibitor treatments. Scale bars, 5 µm. (**i**) Statistical analysis of the volume occupied by NuMA at spindle poles. (**j**) Fluorescence microscopy analysis of Galectin-3 distribution in metaphase cells after DMSO or glycosylation inhibitor treatments. Scale bars, 5 µm. (**k**) Statistical analysis of the intensity of Galectin-3 fluorescence at spindle poles over cytoplasmic Galectin-3 fluorescence intensity. (**l**) Western blot detection of NuMA (*Top blot*) and Galectin-3 (*Bottom blot, black arrowhead*) after the immunoprecipitation of NuMA from mitotic extracts, treated with DMSO or Ac-5SGlcNAc. Input, 10% of total cell lysate. Detailed information about quantifications is available in Supplementary Information.

To further investigate the molecular mechanism of Galectin-3 function, we looked for its protein interactors. We identified a small number of candidates via mass spectrometry that co-immunoprecipitated with Galectin-3 in metaphase enriched cell lysates (Supplementary Fig. S5). Since Galectin-3 is best characterized as a lectin, we focused our analysis on glycoproteins. NuMA (Nuclear Mitotic Apparatus protein) interacts with MT (−)-ends after nuclear breakdown and forms a lattice that stabilizes and strengthens radial spindle MT arrays. NuMA was recently reported to be glycosylated in mitotic cells and it co-immunoprecipitated with Galectin-3 in our assays, making NuMA an excellent candidate interactor of Galectin-3^8, 21^. First, we observed that transfected form of NuMA-GFP and Galectin-3 colocalize at spindle poles in metaphase HeLa cells, and *vice versa* (Fig. 4a). Second, their association was analysed more thoroughly by immunoprecipitation followed by Western blot. We found that Galectin-3 co-immunoprecipitated with NuMA, and *vice versa* (Fig. 4b). We next tested whether the Galectin-3/NuMA association depends on Galectin-3 glycan recognition by performing Galectin-3 immunoprecipitation in the presence of sugar agonists (lactose and galactose) or sugar for which it has no affinity (glucose)^32^ (Fig. 4c). Co-immunoprecipitations in the presence of glucose had no effect, but NuMA’s association with Galectin-3 was abolished when lactose or galactose were added, suggesting that their association is a glycan-dependent interaction. To test the importance of Galectin-3 lectin activity, we analysed the behaviour of sugar binding incompetent variant form and consequences on mitosis. Arg186 is particularly required for sugar binding of the Galectin-3’s CRD domain. Substitution of Arg186 by serine (R186S) has been previously reported to impair Galectin-3 binding to common glycoproteins, whereas the mutation of a close amino acid, i.e. substitution of Gly182 by alanine (G182A), has little effect on CRD activity^32, 33^. Endogenous Galectin-3 was depleted using CRISPR-Cas9 strategy (Supplementary Fig. S6), and wt Galectin-3-GFP, Galectin-3(R186S)-GFP and Galectin-3(G182A)-GFP constructs were introduced by transient transfection in Galectin-3-depleted Hela cells. Removal of Galectin-3 using CRISPR-Cas9 strategy caused an increase of multipolar mitoses in comparison with control CRISPR cells (Fig. 4d,e), as in Galectin-3 siRNA transfection experiments (Fig. 2a,b). While the expression of Galectin-GFP or Galectin-3(G182A)-GFP constructs rescued a bipolar state, Galectin-3(R186S)-GFP transfection did not (Fig. 4d,e), showing that Galectin-3 CRD activity is required for Galectin-3 function in metaphase. Moreover, Galectin-3(R186S)-GFP levels at spindle pole were decreased (Fig. 4d and f), suggesting that glycan binding helps recruit Galectin-3 to the spindle pole. This observation was corroborated by biochemical analysis. NuMA failed to co-immunoprecipitate with Galectin-3(R186S)-GFP form (Fig. 4g). These data indicate that Galectin-3 lectin activity is required to bind NuMA and to promote proper metaphase.

We next asked which kind of glycan chains mediates Galectin-3 association with NuMA. HeLa cells were treated with different glycosylation inhibitors: *N*-glycosylation (swainsonine), GalNAc-type of *O*-glycosylation (GalNAcα-*O*-benzyl) or GlcNAc-type of *O*-glycosylation (Ac-5SGlcNAc)^34^. NuMA or Galectin-3 localization was only disturbed after GlcNAc-type *O*-glycosylation inhibition, with significant spreading of NuMA along spindle MTs (Fig. 4h,i) and decreased Galectin-3 enrichment at spindle poles (Fig. 4j,k). In addition, NuMA and Galectin-3 no longer co-immunoprecipitated in Ac-5SGlcNAc-treated cells (Fig. 4l). Together, these results suggest that the NuMA/Galectin-3 association occurs through an *O*-GlcNAc modification and that it is important for localizing both proteins at the spindle pole.

### Galectin-3 and NuMA interact at spindle poles through an *O*-GlcNAc modification of NuMA

**Figure 5:**
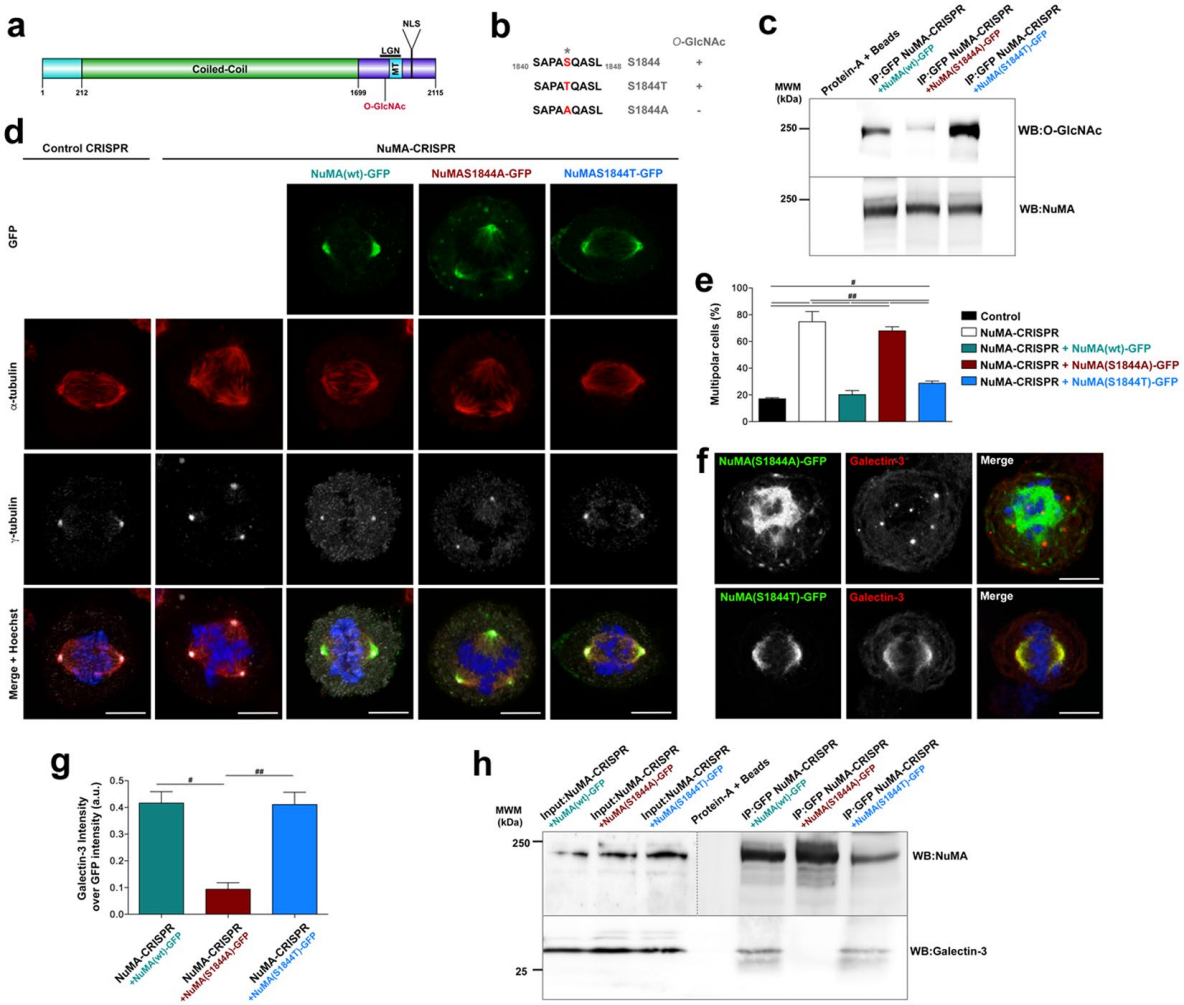
Galectin-3 and NuMA interaction relies on *O*-GlcNAc modification of NuMA. (**a**) Schemes showing NuMA functional domains along its amino acid sequence: in light blue the N-terminal globular domain, in green the central coiled-coil domain and in dark blue the C-terminal globular domain (based on UniprotKB_Q14980). (**b**) Schemes showing the point mutations generated at NuMA *O*-GlcNAc glycosylation site. (**c**) Western blot analysis of NuMA (*Bottom blot*) and O-GlcNAc motifs (*Top blot*) after the immunoprecipitation of GFP from mitotic extracts of NuMA-depleted cells using CRISPR-Cas9 strategy, and transfected with NuMA-GFP constructs. (**d**) Confocal microscopy analysis of α-tubulin and γ-tubulin in control and NuMA-depleted cells using CRISPR-Cas9 strategy, after transfection of NuMA-3-GFP, NuMA-S1844A-GFP or NuMA-S1844T-GFP plasmids. Scale bars, 5 µm. (**e**) Statistical analyses of the number of multipolar cells after transfection of NuMA-GFP constructs in control or NuMA-depleted cells using CRISPR-Cas9 strategy. (**f**) Confocal microscopy analysis of mutated forms of NuMA and Galectin-3 in metaphase cells. Scale bars, 5 µm. Nuclei were detected by Hoechst 33342 staining (*blue*). (**g**) Statistical analysis of the Galectin-3 intensity over NuMA intensity after transfection of NuMA-GFP constructs in NuMA-depleted cells using CRISPR-Cas9 strategy. (**h**) Western blot analysis of NuMA (*Top blot*) and Galectin-3 (*Bottom blot*) after the immunoprecipitation of GFP from mitotic extracts of NuMA CRISPR KO cells transfected with NuMA-GFP constructs. Input, 10% of total cell lysate.

A single *O*-GlcNAc modification site on Ser^1844^ has been previously reported for NuMA^8^ (Fig. 5a). To test whether this specific glycosylation site is implicated in NuMA interaction with Galectin-3, we tested two GFP-tagged mutants forms of NuMA: (i) a glycosylation-blocked mutant form, where the serine is substituted with an alanine and which cannot be *O*-GlcNAc-glycosylated (NuMA-S1844A-GFP), and as a control (ii) a glycosylation-permissive mutant form, which can still be *O*-GlcNAc-glycosylated, by substituting the serine with a threonine (NuMA-S1844T-GFP) (Fig. 5b,c). Endogenous NuMA was depleted using CRISPR-Cas9 strategy (Supplementary Fig. S7), and wt NuMA-GFP, NuMA(S1844A)-GFP and NuMA(S1844T)-GFP constructs were introduced by transient transfection in NuMA-depleted Hela cells. Removal of NuMA using CRISPR-Cas9 strategy caused an increase of multipolar mitoses in comparison with control CRISPR cells (Fig. 5d,e). While the expression of NuMA-GFP or NuMA(S1844T)-GFP constructs rescued a bipolar state, NuMA(S1844A)-GFP transfected cells displayed an increase in multipolar mitoses similar to NuMA-depleted cells (Fig. 5d,e), indicating that indicating that NuMA glycosylation is important for spindle pole formation and stability. Moreover, the glycosylation mutant form was sufficient to induce the formation of cytosolic NuMA foci (Fig. 5f; Supplementary Fig. S8a–c). The NuMA glycosylation mutant was also defective in Galectin-3 recruitment to spindle poles (Fig. 5f,g). We next tested whether the Galectin-3/NuMA interaction depends on NuMA glycosylation state by performing immunoprecipitation. We found that Galectin-3 co-immunoprecipitated with NuMA-GFP and NuMA(S1844T)-GFP, but not with NuMA(S1844A)-GFP (Fig. 5h), showing that Galectin-3 associates with NuMA through its glycan chains. Our results reveal that Galectin-3 interacts with NuMA at the spindle pole through *O*-GlcNAc modifications and that *O*-GlcNAc modification of NuMA is required for its own focusing, as well as Galectin-3 positioning, at the spindle pole during metaphase.

### Galectin-3 acts to organize NuMA at the spindle pole

**Figure 6:**
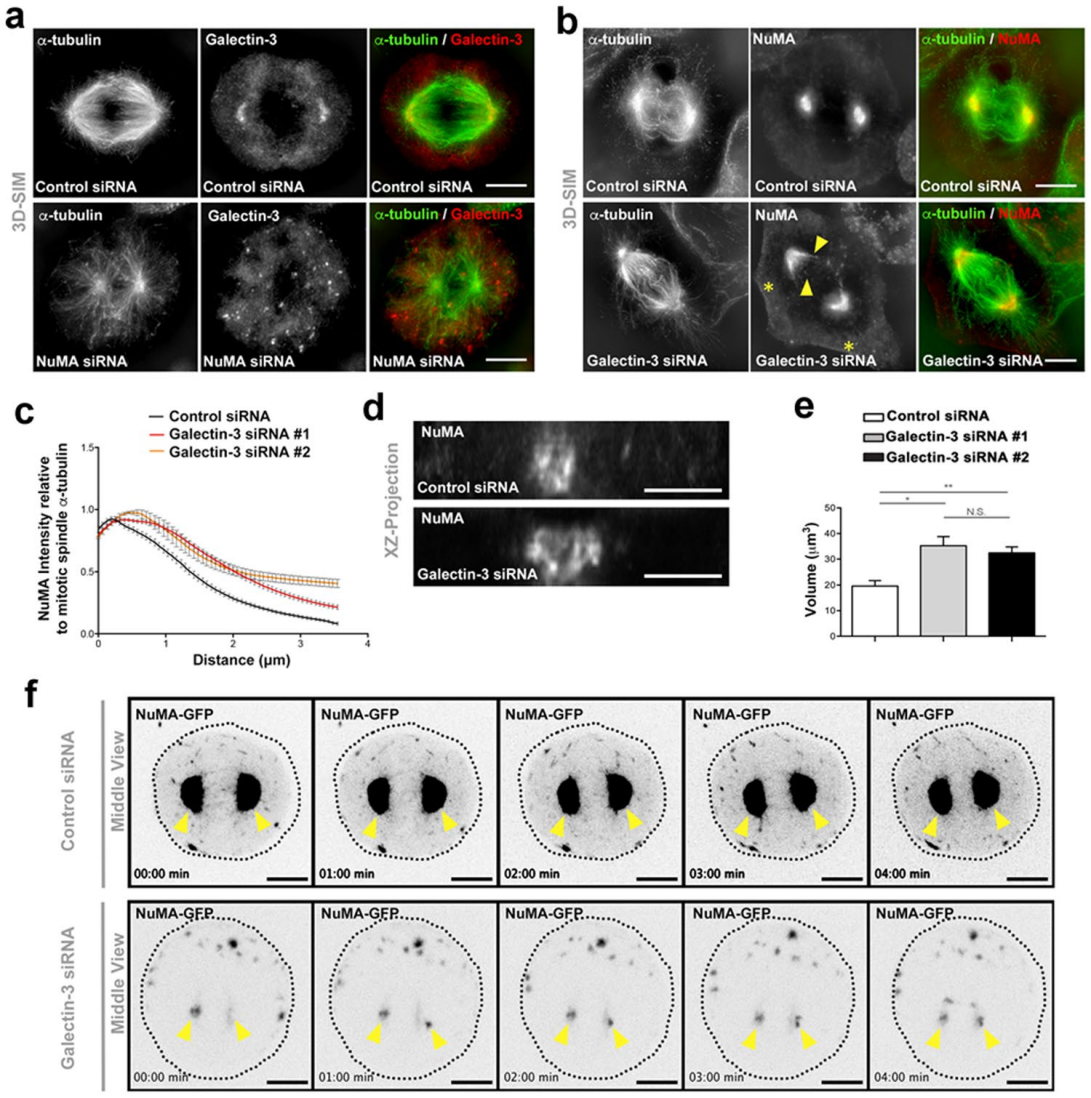
Galectin-3 acts in the organisation of NuMA at the spindle pole. (**a**) 3D-SIM microscopy analysis of α-tubulin (*green*) and Galectin-3 (*red*) distribution in control (*Control siRNA*, *upper panel*) and NuMA-silenced (*NuMA siRNA*, *lower panel*) Hela cells. Deprivation of NuMA leads to the loss of Galectin-3 recruitment at spindle poles and perturbation of mitotic spindles. Scale bars, 5 µm. (**b**) 3D-SIM microscopy analysis of α-tubulin (*green*) and NuMA (*red*) distribution in control (*Control siRNA*, *upper panel*) and Galectin-3 silenced (*Galectin-3 siRNA*, *lower panel*) Hela cells. NuMA is no longer focused at spindle poles and instead spreads along aberrant mitotic spindles (*arrowheads*) in the absence of Galectin-3. In addition, a cortical NuMA signal appears in Galectin-3 siRNA cells (*stars*). Scale bars, 5 µm. (**c**) Statistical analysis of the intensity of NuMA fluorescence over α-tubulin fluorescence intensity along the mitotic spindle in control (*Control siRNA*, *black line*) and Galectin-3-depleted (*Galectin-3 siRNA#1*, *red line and Galectin-3 siRNA#2, orange line*) metaphase Hela cells. *n* (_Control siRNA_) = 24 cells; *n* (_Galectin-3 siRNA#1_) = 24 cells; *n* (_Galectin-3 siRNA#2_) = 24 cells. (**d**) Z-projection of spindle poles immunostained for NuMA in control (*Control siRNA*, *upper panel*) and Galectin-3-depleted (*Galectin-3 siRNA, lower panel*) metaphase Hela cells. (**e**) Statistical analysis of the volume occupied by NuMA at the spindle pole in control (*Control siRNA, white bar*) and Galectin-3-depleted (*Galectin-3 siRNA#1*, *grey bar* and *Galectin-3 siRNA#2, black bar*) metaphase Hela cells. (**f**) Spinning-disc videomicroscopy analysis of NuMA-GFP localization in control (*Control siRNA*, *upper panels*) and Galectin-3-silenced (*Galectin-3 siRNA*, *lower panels*) metaphase Hela cells. Middle views going through the spindle poles (*yellow arrowheads*) are shown. Time frames from 0 to 4 min are presented.

We next evaluated a functional interaction between Galectin-3 and NuMA. We found that siRNA silencing of NuMA displaced Galectin-3 from the spindle pole in HeLa cells (Supplementary Fig. S9; Fig. 6a). Conversely, siRNA reduction of Galectin-3 led to NuMA mislocalization (Fig. 6b). NuMA normally localizes in a concise ring at mitotic poles and dimly at the plasma membrane in control cells (Fig. 6b; Supplementary Fig. S10a,b). However, Galectin-3 silencing leads to NuMA spreading along spindle MTs (Fig. 6b,c). In addition, the NuMA lattice at the spindle was deformed (Fig. 6d). Whereas the NuMA ring occupied a mean volume of 19.6 ± 2.1 µm^3^ in control cells, it increased to 35.2 ± 3.6 and 32.5 ± 2.3 µm^3^ in Galectin-3-depleted cells (Fig. 6e). Defective NuMA localization was further analysed with live cell imaging. We observed that NuMA-GFP shuttled between the spindle poles and the cell cortex in control cells (Fig. 6f, Supplementary movie 1), but larger foci of NuMA (as observed previously with GFP-mutant version of NuMA in NuMA KO cells, Supplementary Fig. S8) and unshaped spindle poles appeared in Galectin-3-depleted cells (Fig. 6f, Supplementary Fig. S11 and Supplementary movie 2). These results show that NuMA and Galectin-3 are functionally interdependent for their spindle pole localization in metaphase cells.

### Galectin-3 participates in spindle pole cohesion and pericentrosomal matrix stabilization

**Figure 7:**
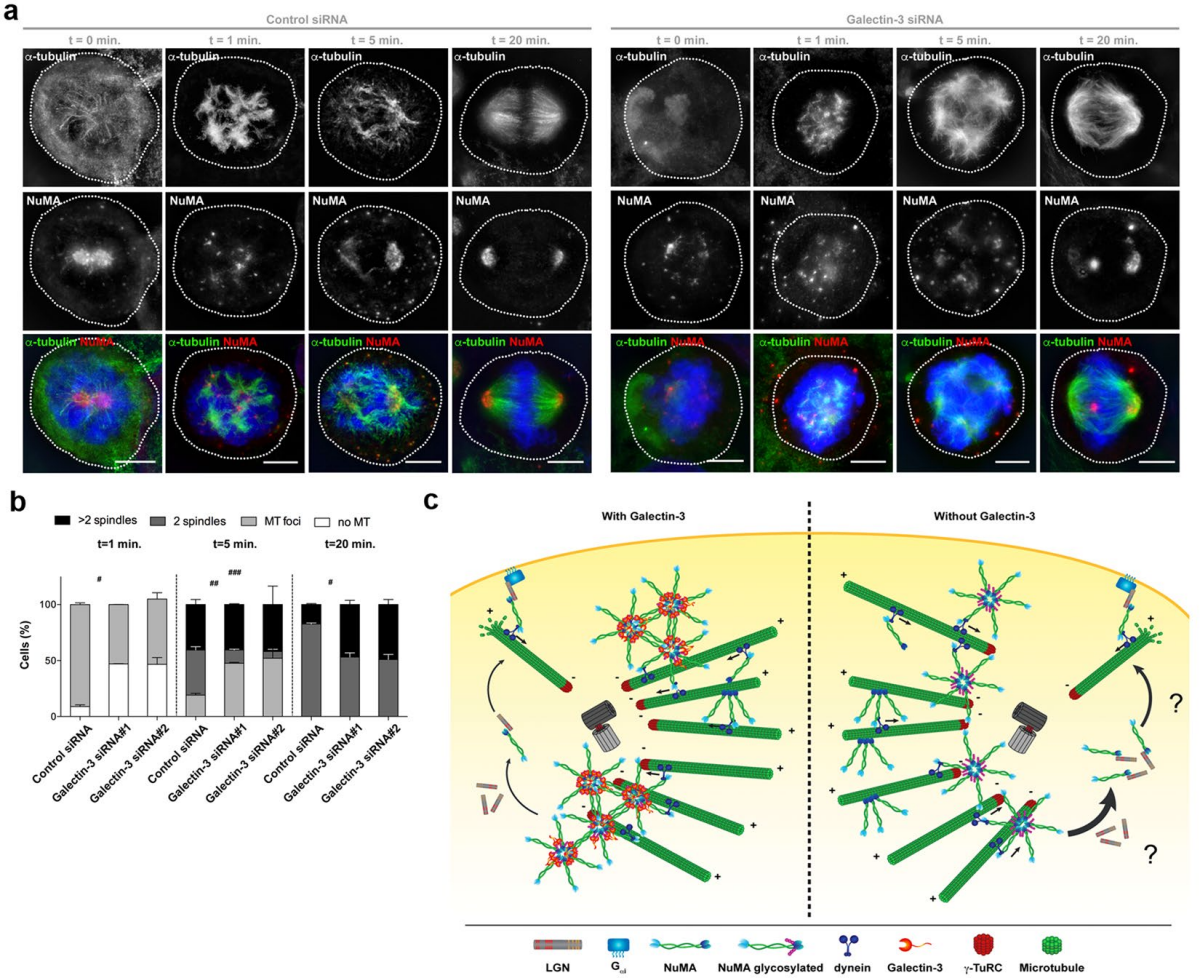
Galectin-3 participates in spindle pole cohesion and pericentrosomal matrix distribution. (**a**) 3D-SIM microscopy analysis of α-tubulin (*green*) and NuMA (*red*) after nocodazole treatment, wash out and MT regrowth for 0, 1, 5 or 20 min in control (*Control siRNA*, *left panels*) or Galectin-3-deprived (*Galectin-3 siRNA*, *right panels*). Nuclei were detected by Hoechst 33342 staining (*blue*). Scale bars, 5 µm. (**b**) Statistical analysis of metaphase morphology during MT regrowth at different time point (from left to right, separated by a doted-line, 1, 5 and 20 min after nocodazole washout/MT regrowth) in control and Galectin-3-depleted HeLa cells. Metaphase morphology has been set in four different categories; no MT (*white*): no MT appeared to be grown; MT foci (*light grey*): MT are organized in *bouquet* originating from a single γ-tubulin/NuMA foci; 2 spindles (*dark grey*): metaphase presented a classical bipolar organization; > 2 spindles (*black*): metaphase presented a multipolar profile. Data are means ± SEM. (**c**) Scheme showing the predicted mechanism of action of Galectin-3 in the stabilization of NuMA complexes and spindle pole cohesion in metaphase epithelial cells. As previously proposed^47^, NuMA oligomerizes through its C-terminal globular domain (*dark blue*) to form radial structures that would interact altogether to form higher structure. NuMA interacts directly with MTs and dynein through its C-terminal globular domain (*dark blue*) and N-terminal region (*light blue*), respectively. The glycosylation (*purple*) is present on the C-terminal globular domain (*dark blue*). As Galectin-3 would recognize the glycosylation on the C-terminal globular domain, we hypothesize that it would facilitate the oligomerization of NuMA. This will contribute to a correct organization of spindle pole (*left panel*). We hypothesize that the absence of Galectin-3 might disturb NuMA oligomerized structures, affecting the structure of the spindle pole (*right panel*). Moreover, perturbation of the oligomerization of NuMA might lead to an increase of NuMA shuttling to the cortex through dynein-based transport and LGN interaction at plasma membrane (*question mark, right panel*). Detailed information about quantifications is available in Supplementary Information.

To study the consequence of the loss of Galectin-3 to spindle and pole formation, we looked at spindle pole reformation after MT depolymerisation by nocodazole treatment and wash out (Fig. 7a). In control cells, small MT arrays form all over at NuMA foci one minute after nocodazole wash out (Fig. 7a, *left panels*). By contrast, Galectin-3-depleted cells show disorganized and fewer MTs and disorganized arrangements (Fig. 7a,b, *right panels*). Five minutes after nocodazole wash out, an unshaped spindle appeared with characteristic astral and interpolar MTs in control cells (Fig. 7a, *left panels*). However, multiple small spindles remained, as well as collection of MTs and NuMA foci in Galectin-3 depleted cells compared to control cells (Fig. 7a,b, *right panels*). Finally, after twenty minutes, control cells displayed normal spindle poles and MT arrays (Fig. 7a,b, *left panels*), but Galectin-3 depleted cells displayed incomplete reformed spindle poles (Fig. 7a,b, *right panels*). We also noticed that Galectin-3-depleted spindles were defective, either improperly shaped or with scattered spindle poles (Fig. 7a, *right panels*). These data suggest that Galectin-3 acts as an organizer of the NuMA network at spindle poles. In addition, the absence of Galectin-3 induces a delay in the gathering of individual MTs in foci, impairing the proper formation of spindle poles and the metaphase plate (Fig. 7c).

## Discussion

Cells have evolved many molecular mechanisms to precisely control the sequence of mitotic events and avoid aneuploidy. Spindle pole integrity is a critical feature that ensures correct DNA segregation. Here we confirm and extend the importance of an unconventional *O*-GlcNAc-glycosylation during metaphase, and identify and characterize the role of the lectin Galectin-3. While most glycosylation occurs in the Golgi apparatus, *O*-GlcNAc-glycosylation takes place in the cytosol under control of *O*-linked *N*-acetylglucosamine transferase (OGT)^35^. Hart and colleagues demonstrated that OGT enzyme and *O*-GlcNAc-modified proteins are enriched at mitotic spindles in HeLa cells, and modulation of OGT expression leads to metaphase and cytokinesis defects^7, 8^. Notably, *O*-GlcNAc modification is critical to the activity of NuMA, a key player in the establishment and the maintenance of spindle poles, i.e. NuMA^21, 36, 37^. In this study, we found that Galectin-3 is a functional partner of NuMA, binding through *O*-GlcNAc moieties during metaphase. This interaction is important for mitosis as it promotes NuMA focusing at spindle poles. Moreover, Galectin-3 silencing mimics the metaphase perturbation observed when OGT expression is reduced^8^. These results suggest a model in which Galectin-3 interacts with glycosylated NuMA to organize the spindle pole (Fig. 7c). At the molecular level, it is well-established that Galectin-3 acts as an oligomerization factor when bound to its ligands, and it mediates the formation of lattices that stabilize or gather many different proteins^38^. For instance, Galectin-3 ensures the polymerization of Hensin, an extracellular matrix protein implicated in epithelial cells differentiation^39^. The existence of an oligomerization factor has been hypothesized to promote the formation of the NuMA matrix, but this factor had not been identified^21^. This work suggests that Galectin-3 could physically bind NuMA and thereby trigger the formation and/or the stabilization of a NuMA lattice at spindle poles (Fig. 7c). NuMA-Galectin-3 association is critical to stabilize the NuMA matrix at spindle poles as well as for MT dynamics at spindles, validating the longstanding hypothesis that post-translational modification of NuMA and an additional NuMA-binding factor promote NuMA lattice ordering at the spindle pole^17^. In addition, the Ser^1844^ *O*-GlcNAc-glycosylation site is located close to the LGN binding domain in the NuMA sequence^40^ (Fig. 3i). Disrupting the Galectin-3/NuMA association induces a weakening of the NuMA lattice at the spindle, but it also causes an increased pool of NuMA to localize to the cell cortex (Fig. 4b; Supplementary Fig. S10). Could *O*-GlcNAc-glycosylation influence NuMA interaction with LGN? We speculate that stabilization of NuMA at the spindle pole by Galectin-3 may also prevent the accumulation NuMA dimers at the cortex by LGN for correct progression through metaphase (Fig. 5b).

Galectin-3 modulates the organization of MT (−)-ends via NuMA at spindle poles, and this may be similar to the recently proposed function of Galectin-3 recently proposed at basal bodies of multiciliated cells^7^. The lectin ensures the recruitment of MTs and consequently the maintenance of the basal body MTOC, through the stabilization of γ-tubulin at basal foot cap^7^. It will be interesting to investigate whether Galectin-3 functions generally in the organization of the MTOC and MT arrays in different types of epithelial cells. Galectin-3 is mostly expressed in epithelia and is considered as a multifunctional protein, due to the diverse cellular functions that it promotes^41^.

Defects in Galectin-3 expression (either loss or over) are correlated with cancer progression and are actually correlated with poor diagnosis^16, 42, 43^. Two roles of Galectin-3 in immune response and neoangiogenesis have been largely described^17, 44^, but prior to this work, a direct role for Galectin-3 in cell division had not been investigated. Although Raz and colleagues showed that the lectin helps modulate cell cycle progression, through indirect repression of cyclin-E and A and inducing cyclin D1 expression in breast cancer cells *in vitro* ^45^. Here we show that Galectin-3 directly participates in proper completion of epithelial mitosis. We were surprised to find that *gal3*−/− mice do not display any obvious phenotypic and morphological abnormalities under physiological conditions and do not spontaneously develop tumors (Fig. 1b)^46^. However, they are sensitive to additional stresses such as tissue damage/loss or genetic mutations. Thus loss of Galectin-3 could sensitize cells to the loss of additional cell division regulators, which could trigger morphogenetic alterations and/or epithelial tumor development.

## Material and Methods

### Materials

Rabbit antibodies directed against Galectin-3 (1:200) and Galectin-1 (1:200) were kindly provided by Dr. H. P. Elsaesser (Philipps University, Marburg, Germany) and Dr. D. N. W. Cooper (University of California, San Francisco, California, USA), respectively. Mouse anti-α-tubulin (T9026; 1:100), anti-acetylated-α-tubulin (T6793; 1:100), anti-γ-tubulin (T6557; 1:200), and rabbit anti-γ-tubulin (T3559; 1:200) antibodies were from Sigma-Aldrich (St Louis, Missouri, USA). Rabbit anti-ninein (ab4447; 1:100), rabbit anti-NuMA (ab109262; 1:200) and mouse anti-*O*-GlcNAc (ab2739; 1/500) antibodies were from Abcam (Cambridge, UK). Mouse anti-dynein intermediate chains, cytoplasmic (MAB1618; 1:100) and rabbit anti-phosphorylated (Ser10) histone H3 (06-570; 1:200) antibodies were from EMD Millipore (Billerica, Massachusetts, USA). Rabbit anti-NuMA (PAB12087; 1:200) antibody was from Abnova (Taipei, Taiwan). Mouse anti-actin (8691001) antibody was from MP Biomedicals (Santa Ana, California, USA). Human anti-CREST (15-235; 1:200) antibody was from Antibodies Incorporated (Sacramento, California, USA). Rabbit anti-pericentrin (PRB432C; 1:100) antibody was from Covance (Princeton, New-Jersey, USA). Mouse anti-GFP antibody (#11.814.460.001, clones 7.1 and 13.1; 1/500) was from Roche (Basel, Switzerland). Alexa-488, -568, or -633 secondary antibodies (1:250), Hoechst 33342 (H3570) and Alexa-647-Phalloidin (A22287; 1:50) were from Life Technologies (Paisley, UK). *O*-linked *N*-acetylglucosamine inhibitor, Ac-5S-GlcNAc^34^ was provided by Dr. D. Vocadlo (Simon Fraser University, Burnaby, Canada). Benzyl 2-acetamido-2-deoxy-α-D-galactopyranoside (GalNAc-α-*O*-Benzyl; B4894), Protein-A/Sepharose beads (P9424), nocodazole (M1404), swainsonine (S9263) and HRP-labelled secondary antibodies were from Sigma-Aldrich.

### Mice and preparation of tissue samples

Mice were housed in EOPS (Environment without Specific Pathogenic Organisms) environment, and handled in accordance with French regulation for animal care. Experimental procedures were performed in accordance with Departmental Director of “Services Vétérinaires de la Préfecture de Police de Paris”. Nephron reduction experimental protocol was approved by the ethical committee of the Paris Descartes University (approval number: A75-15-34).

2-month-old *Galectin-3*−/− 129/Sv mice^46^ and 129/Sv wild-type littermates were subjected to 75% nephrectomy (*Nx*) or sham operation (*Control*), as previously described^22^. After surgery, mice were fed a defined diet containing 30% casein and 0.5% sodium. Mice were sacrificed by cervical dislocation 90 days after surgery and weighed. Plasma samples were collected at the time of sacrifice and plasma creatinine was measured using Konelab Analyzer (Thermo Fisher Scientific, Waltham, MA, USA). Kidneys were harvested and weighed prior to analyses.

For immunohistochemistry, half-kidneys from 7 sham-operated *wt*, 5 sham-operated *gal3*−/−, 23 Nx *wt* and 20 Nx *gal3*−/− mice were fixed by successive 1 hour incubations in cold 70, 90, and 100% methanol solutions and then paraffin-embedded. Half kidneys were fixed overnight in 4% paraformaldehyde. The tissues were then processed for paraffin embedding as previously described^6^. Paraffin sections were prepared on poly-L-lysine hand-coated microscope slides for analyses. For cell proliferation analyses, 200 μg EdU (Thermo Fischer Scientific) in 100 μl sterile PBS was delivered 4 h prior mouse sacrifice by intraperitoneal injection.

### Histological and immunohistological analyses

For histology, 5 µm paraffin sections were stained with Hematoxylin and Eosin (H&E) as previously described^6^. Mosaics of H&E-stained kidney sections were created under a 10x lens using a motorised Zeiss Axiovert200 microscope equiped with a color camera AxioCam HRc and Zeiss AxioVision software (Zeiss, Oberkochen, Germany). Zooms were taken under a 40x lens. The degree of tubular dilation was automatically quantified using a Nikon digital camera Dx/m/1200 and NIS software (Laboratory Imaging Ltd). Ten randomly selected microscopic fields (X200) were scored. Surgical scars were excluded from analysis.

For immunostaining, 8μm (cilium and centrosome analyses) or 30 µm (mitosis analyses) paraffin sections were dewaxed in a xylene bath and rehydrated once in isopropanol and in increasing ethanol solutions. Sections were saturated in 10% goat serum (Dako, Carpinteria, California, USA) for 30 min. Primary antibodies incubations were performed at 4 °C for 12 h in 10% goat serum solution. Secondary antibody incubations were done at room temperature for 2 hours in 10% goat serum solution. Tissue sections were mounted in Mowiol 4-88 (EMD Millipore) solution. To label proliferative cells, paraffin sections were stained with the Click-iT Edu Alexa Fluor 488 Imaging kit (Thermo Fisher Scientific).

### Cell culture

HeLa cells were maintained in culture in DMEM High-Glucose (11965-092; Life Technologies) supplemented with 10% FBS and 1% Penicillin-Streptomycin (Life Technologies). To investigate metaphase plate position, the cells were fixed for 1 min in 0.025% glutaraldehyde, 0.05% Triton X-100 followed by 10 min in 1% glutaraldehyde and 45 min in 0.5 mg/mL NaBH4 all in cytoskeletal buffer^48^. For other immunostainings, the cells were fixed with pre-cooled 100% methanol (−20 °C) for 5 min. The cells were then processed for immunofluorescence microscopy as previously described^14^ and mounted in Mowiol 488 (EMD Millipore).

Transfections were carried out using Lipofectamine 2000 reagent (Life Technologies) following the manufacturer’s recommendations. Galectin-3 or NuMA reduction were performed by transfecting a mix of siRNA duplexes directed against human Galectin-3 (gal3 siRNA #1: 5′-AUAAGGCACAAUCAGUGGC-3′/5′-GCCACUGAUUGUGCCUUAU-3′, gal3 siRNA #2: 5′-ACCCAAACCCUCAAGGAUGTT-3′/5′-CCAUCUUCUGGACCAGCCAA-3′), or human NuMA1 (NuMA siRNA #1: 5′-CCUCACCGAGAAGGAUGCACAGAUA-3′/5′-UAUCAGUGCAUCCUUCUCGGUGAGG-3′, NuMA siRNA #2: 5′-CAAAGAGCUGCGAGCUGAAGCUGAA-3′/5′-UUCAGCUUCAGCUCGCAGCUCUUUG-3′) (LifeTechnologies). Control transfections were performed with luciferase siRNA (Dharmacon, GE-healthcare, Chalfont St. Giles, UK). Phenotypes of Galectin-3 or NuMA reduction was analyzed 48 to 72 h later.

For rescue experiments, Galectin-3 or NuMA depletion were performed by transfecting Galectin-3 CRISPR/Cas9 Knockout or NuMA CRISPR/Cas9 Knockout plasmids purchased from Santa Cruz (Dallas, Texas, USA). CRISPR/Cas9 plasmids were co-transfected with puromycin resistance gene coding plasmid. Co-transfected cells were selected in DMEM supplemented with 1 μg/ml puromycin (Invivogen, San Diego, CA, USA).

### Plasmids

Galectin-3-GFP, Galectin-3-R186S-GFP and Galectin-3-G182A-GFP were generated as previously described^33^. NuMA-GFP construct was purchased from Addgene (Dr. M. Mancini (Baylor College of Medicine, Texas, USA). Mutated forms of NuMA-GFP were generated by site-directed mutagenesis using a Phusion-site directed mutagenesis kit (Thermo Scientific) with forward primers for the S1844T form 5′-GCTCCTGCTACTCAGGCTAGCCTG-3′ and for the S1844A form 5′-GCTCCTGCTGCTCAGGCTAGCCTG-3′ and as reverse primer 5′-AGCAGGAGCAGACCGCGTGCTGTAG-3′.

### Drug Treatment

For microtubule regrowth experiments, HeLa cells were incubated in 10 ng/ml nocodazole for 4 h at 37 °C, followed by a 30 min at 4 °C. Microtubule regrowth was initiated by with pre-warmed complete media and incubation at 37 °C for several time points. Cells were then analyzedafter fixation in −20 °C pre-cooled methanol for 5 min. To inhibit *O*-linked *N*-acetylgalactosamine addition, cells were treated with 4 mM GalNAc-α-*O*-benzyl for 48 h. To inhibit *N*-linked glycosylation, cells were treated with 10 µg/ml swainsonine for 24 h. To inhibit *O*-linked *N*-actylglucosamine addition, cells were treated with 50 or 100 µM Ac-5S-GlcNAc for 48 h.

### Biochemistry

Cell lysates were prepared as previously described^14^. For immunoprecipitation, cell extracts were prepared from enriched metaphase cells by mitotic shake-off. Mitotic cell lysates were precleared by incubation with protein-A/Sepharose beads, and then antibodies were applied. Immunoprecipitation of Galectin-1 and incubation of protein-A coated beads alone served as controls. Immunocomplexes were recovered by addition of Protein-A/Sepharose beads overnight at 4 °C, followed by three washes in PBS. Finally, the beads were resuspended in Laemmli buffer for Western blot. For the identification of Galectin-3 binding proteins, beads were submitted to proteolytic digestion and the peptides were analyzed by mass spectrometry (ESI-LTQ-Orbitrap spectrometer) coupled with nano LC chromatographic separation system (Proxeon) in the IJM Proteomic facility. For immunoprecipitation in presence of sugar agonists, glucose, lactose or galactose was added to the lysis buffer to a final concentration of 300, 100 and 300 mM, respectively. Samples were loaded in NuPAGE precast polyacrylamide gels (Thermo Scientific). Primary antibodies were detected using HRP-conjugated secondary antibodies and SuperSignal™ West Femto Maximum Sensitivity Substrate (Thermo Scientific), visualized on a ImageQuant LAS4000 (GE-Healthcare). Signal quantification was performed using ImageJ.

### Image acquisition

Immunofluorescent images were acquired on a Zeiss Axio Imager.Z1 microscope (Zeiss) equipped with an AxioCam digital camera using a 100x NA 1.4 objective. When applied, deconvolution was performed as previously described^49^. Confocal image sections were acquired on Leica TCS SP5 microscope (Leica Microsystems, Deerfield, Illinois, USA) using a 63x NA 1.4 and 100x NA 1.4 lenses from the Imagoseine plateform (IJM). 3D-SIM images were acquired on the Nikon N-SIM system using a 100x APO TIRF NA 1.49 objective in the NIKON Imaging Center at the Curie Institute (Paris, France). The images reconstruction based on the Gustafsson methods^50^ were performed on the NIS-Elements Ar software. Videomicrosocopy were performed with a spinning-disk confocal set-up on a Nikon TiE inverted microscope equiped with a CoolSNAP HQ2 camera operating under Metamorph software. Images were then adjusted for brightness and contrast using ImagJ software.

For ciliary length measurement, primary cilia were detected using acetylated-α-tubulin immunostaining. 3D reconstruction of confocal Z-stacks acquired was performed and ciliary length was measured using the filament option of Imaris Software.

For the enrichment of Galectin-3 at the spindle, ImagJ was used to manually measure the total pixel intensity of Galectin-3 at the spindle pole, which was reported to the total pixel intensity of Galectin-3 in the cytoplasm. The measurement of NuMA volume along the spindle was performed on ImageJ as previously described^51^. Spindle pole was manually isolated using α-tubulin staining. Line scan was performed for each isolated spindle for α-tubulin and the corresponding marker analyzed. Volume analysis was performed using 3D Object Counter plugin^52^ on ImageJ.

### Statistical analyses

Statistical analyses have been performed using Prism (GraphPad software, La Jolla, CA, USA).

## Electronic supplementary material

Supplementary movie 1

Supplementary movie 2

Supplementary Information
