# Supplementary material for: Spindle pole cohesion requires glycosylation-mediated localization of NuMA
Source: Sci Rep. 2017 May 3;7:1474. doi: 10.1038/s41598-017-01614-6 (PMC5431095; doi:10.1038/s41598-017-01614-6)
Supplement: Supplementary file 3 — Supplementary Information [file 41598_2017_1614_MOESM3_ESM.pdf]

Spindle pole cohesion requires glycosylation-mediated localization of NuMA

Jérémy Magescas, Lucie Sengmanivong, Amandine Viau, Adeline Mayeux, Tien Dang, Martine Burtin, Ulf J. Nilsson, Hakon Leffler, Françoise Poirier, Fabiola Terzi and Delphine Delacour

Supplementary Information Guide

Supplementary Information: Additional information for Figure legends 1, 2, 4, 5, 6 and 7

Supplementary movies 1 and 2

Supplementary Figures 1 to 11

Supplementary Information: Figure legends 1, 2, 4, 5, 6 and 7

Figure 1:

b-c, *N* ($_{\text{Control wt}}$) = 3 mice; *N* ($_{\text{Control } gal3\text{-/-}}$) = 2 mice; *N* ($_{\text{Nx wt}}$) = 13 mice; *N* ($_{\text{Nx } gal3\text{-/-}}$) = 15 mice. ***d***, *N* ($_{\text{Control wt}}$) = 7 mice, *N* ($_{\text{Control } gal3\text{-/-}}$) = 5 mice, *N* ($_{\text{Nx wt}}$) = 23 mice, *N* ($_{\text{Nx } gal3\text{-/-}}$) = 20 mice. Mean kidney weight over mouse weight $_{\text{(wt control)}}$ = 6.68±0.28 mg/g, mean kidney weight over mouse weight $_{(gal3\text{-/- control})}$ = 6.21±0.19 mg/g, mean kidney weight over mouse weight $_{\text{(wt nephron reduction)}}$ = 6.83±0.24 mg/g, mean kidney weight over mouse weight $_{(gal3\text{-/- nephron reduction})}$ = 8.48±0.35 mg/g. Ordinary one-way ANOVA followed by Tukey-Kramer test. #*P*-value ($_{\text{Control wt vs. Nx } gal3\text{-/-}}$) = 0.0096; ##*P*-value ($_{\text{Control } gal3\text{-/- vs. Nx } gal3\text{-/-}}$)= 0.0034; ###*P*-value ($_{\text{Nx wt vs. Nx } gal3\text{-/-}}$) = 0.0004. ***e***, *N* ($_{\text{Control wt}}$) = 3 mice; *N* ($_{\text{Nx wt}}$) = 13; *N* ($_{\text{Control } gal3\text{-/-}}$) = 2; *N* ($_{\text{Nx } gal3\text{-/-}}$) = 15. Mean tubular lesion score $_{\text{(wt control)}}$ = 1.94±0.26 %, $_{(gal3\text{-/- control})}$ = 3.19±0.60 %, $_{\text{(wt nephron reduction)}}$ = 7.77±0.63 %, $_{(gal3\text{-/- nephron reduction})}$ = 12.71±2.36 %. Ordinary one-way ANOVA followed by Tukey-Kramer test. #*P*-value ($_{\text{Control wt vs. Nx wt}}$) = 0.012; ##*P*-value ($_{\text{Control wt vs. Nx } gal3\text{-/-}}$) = 0.0091; ###*P*-value ($_{\text{Control } gal3\text{-/- vs. Nx wt}}$) = 0.044; ####*P*-value ($_{\text{Control } gal3\text{-/- vs. Nx } gal3\text{-/-}}$) = 0.036; #####*P*-value ($_{\text{Nx wt vs. Nx } gal3\text{-/-}}$) = 0.021. ***f***, *N* ($_{\text{Control wt}}$) = 3 mice; *N* ($_{\text{Nx wt}}$) = 2; *N* ($_{\text{Control } gal3\text{-/-}}$) = 8; *N* ($_{\text{Nx } gal3\text{-/-}}$) = 9. Mean serum creatinine $_{\text{(wt control)}}$ = 5.33±0.28, $_{(gal3\text{-/- control})}$ = 5.05±0.25, $_{\text{(wt nephron reduction)}}$ = 8.50±0.58, $_{(gal3\text{-/- nephron reduction})}$ = 15.12±1.55. Ordinary one-way ANOVA followed by Tukey-Kramer test. #*P*-value ($_{\text{Control wt vs. Nx } gal3\text{-/-}}$) = 0.0008; ##*P*-value ($_{\text{Control } gal3\text{-/- vs. Nx } gal3\text{-/-}}$) = 0.0021; ###*P*-value ($_{\text{Nx wt vs. Nx } gal3\text{-/-}}$) = 0.0054. ***g***, *N* ($_{\text{Control wt}}$) = 3 mice; *N* ($_{\text{Nx wt}}$) = 2; *N* ($_{\text{Control } gal3\text{-/-}}$) = 8; *N* ($_{\text{Nx } gal3\text{-/-}}$) = 9. Mean proliferative index $_{\text{(wt control)}}$ = 0.23±0.071, $_{(gal3\text{-/- control})}$ = 0.18±0.048, $_{\text{(wt nephron reduction)}}$ = 0.83±0.15, $_{(gal3\text{-/- nephron reduction})}$ = 1.66±0.24. Ordinary one-way ANOVA followed by uncorrected Fisher's LSD. #*P*-value ($_{\text{Control wt vs. Nx wt}}$) = 0.0307; ##*P*-value ($_{\text{Control wt vs. Nx } gal3\text{-/-}}$) < 0.0001; ###*P*-value ($_{\text{Control } gal3\text{-/- vs. Nx wt}}$) = 0.0337; ####*P*-value ($_{\text{Control } gal3\text{-/- vs. Nx } gal3\text{-/-}}$) = 0.0001; #####*P*-value ($_{\text{Nx wt vs. Nx } gal3\text{-/-}}$) = 0.0024. ***i***, *N* ($_{\text{Control wt}}$) = 4 mice, *N* ($_{\text{Control } gal3\text{-/-}}$) = 4, *N* ($_{\text{Nx wt}}$) = 4, *N* ($_{\text{Nx } gal3\text{-/-}}$) = 4, n ($_{\text{Control wt}}$) = 453 cilia, n ($_{\text{Control } gal3\text{-/-}}$) = 415, n ($_{\text{Nx wt}}$) = 308, n ($_{\text{Nx } gal3\text{-/-}}$) = 880. Mean primary cilium length $_{\text{(wt control)}}$ = 2.7±0.043 µm, $_{(gal3\text{-/- control})}$ = 2.9±0.058 µm, $_{\text{(wt nephron reduction)}}$ = 4.0±0.12 µm, $_{(gal3\text{-/- nephron reduction})}$ = 9.4±0.24 µm. Ordinary one-way ANOVA followed by Tukey-Kramer test. #*P*-value ($_{\text{Control wt vs. Nx wt}}$) = 0.020; ##*P*-value ($_{\text{Control wt vs. Nx } gal3\text{-/-}}$) < 0.0001; ###*P*-value ($_{\text{Control } gal3\text{-/- vs. Nx wt}}$) = 0.0142; ####*P*-value ($_{\text{Control } gal3\text{-/- vs. Nx } gal3\text{-/-}}$) < 0.0001; #####*P*-value ($_{\text{Nx wt vs. Nx } gal3\text{-/-}}$) < 0.0001. ***l***, *n* ($_{\text{Control wt}}$) = 7 cells in 4 mice; *n* ($_{\text{Nx wt}}$) = 15 cells in 3 mice; *n* ($_{\text{Control } gal3\text{-/-}}$) = 5 cells in 3 mice; *n* ($_{\text{Nx } gal3\text{-/-}}$) = 43 cells in 3 mice. Mean abnormal metaphase morphology $_{\text{(wt control)}}$ = 8.33±8.33 %, $_{(gal3\text{-/- control})}$ = 0.0±0.0 %, $_{\text{(wt nephron reduction)}}$ = 4.44±4.44 %, $_{(gal3\text{-/- nephron reduction})}$ = 51.03±5.76 %. Ordinary one-way ANOVA followed

by Tukey-Kramer test. #*P*-value ($_{\text{Control wt vs. Nx } gal3\text{-/-}}$) = 0.0036; ##*P*-value ($_{\text{Control } gal3\text{-/- vs. Nx } gal3\text{-/-}}$) = 0.0017; #####*P*-value ($_{\text{Nx wt vs. Nx } gal3\text{-/-}}$) = 0.0032.

Figure 2:

b, Mean multipolar cells ($_{\text{Control siRNA}}$) = 6.95±2.20 %, ($_{\text{Galectin-3 siRNA \#1}}$) = 23.14±3.53 % and ($_{\text{Galectin-3 siRNA \#2}}$) = 23.28±2.51 %. Ordinary one-way ANOVA followed by Tukey-Kramer test. ##*P*-value ($_{\text{Control siRNA vs. Galectin-3 siRNA\#1}}$) =0.007; #*P*-value ($_{\text{Control siRNA vs. Galectin-3 siRNA \#2}}$) = 0.0066. ***d***, Mean acentrosomal spindle(s) cells ($_{\text{Control siRNA}}$) = 32.42±3.16 %, ($_{\text{Galectin-3 siRNA \#1}}$) = 71.48±13.03 %, ($_{\text{Galectin-3 siRNA \#2}}$) = 73.18±11.76 %. Mean centrosomal spindle(s) cells ($_{\text{Control siRNA}}$) = 67.58±3.16 %, ($_{\text{Galectin-3 siRNA \#1}}$) = 28.52±13.03 %, ($_{\text{Galectin-3 siRNA \#2}}$) = 26.82±11.76 %. Ordinary two-way ANOVA followed by Tukey-Kramer test. #*P*-value ($_{\text{Control siRNA vs. Galectin-3 siRNA\#1}}$) =0.0015; ##*P*-value ($_{\text{Control siRNA vs. Galectin-3 siRNA \#2}}$) =0.0011.

Figure 4:

e, *N* ($_{\text{Control cells}}$) = 420 cells, *N* ($_{\text{Galectin-3 CRISPR}}$) = 475, *N* ($_{\text{Galectin-3-GFP}}$) =616, *N* ($_{\text{Galectin-3-R186S-GFP}}$) = 526, *N* ($_{\text{Galectin-3-G182A-GFP}}$) = 527. Mean multipolar cells ($_{\text{Control cells}}$) = 9.2±0.8 %, ($_{\text{Galectin-3 CRISPR}}$) = 75.4±2.3 %, ($_{\text{Galectin-3-GFP}}$) = 26.6±2.7 %, ($_{\text{Galectin-3-R186S-GFP}}$) = 69.9±1.1 %, ($_{\text{Galectin-3-G182A-GFP}}$) = 29.5±3.0 %. Ordinary one-way ANOVA followed by Tukey-Kramer test. #*P*-value = 0.008. Data are means ± SEM. ***f***, *N* ($_{\text{Galectin-3-GFP}}$) =15 cells, *N* ($_{\text{Galectin-3-R186S-GFP}}$) = 17, *N* ($_{\text{Galectin-3-G182A-GFP}}$) = 17. Mean Galectin-3 intensity ratio ($_{\text{Galectin-3-GFP}}$) = 3.77±0.19, ($_{\text{Galectin-3-R186S-GFP}}$) = 2.32±0.15, ($_{\text{Galectin-3-G182A-GFP}}$) = 3.72±0.49. Ordinary one-way ANOVA followed by Tukey-Kramer test. #*P*-value = 0.0015; ##*P*-value = 0.0004; ###*P*-value < 0.0001. Data are means ± SEM. ***i***, *N* ($_{\text{DMSO}}$) = 25 cells, *N* ($_{\text{Swainsonine}}$) = 25, *N* ($_{\text{GalNAc-}\alpha\text{-}O\text{-benzyl}}$) = 25, *N* ($_{\text{Ac-5SGlcNAc}}$) = 25. T-test analysis, #*P*-value < 0.0001; ##*P*-value < 0.0001; ###*P*-value < 0.0001. Data are means ± SEM. ***k***, *N* ($_{\text{DMSO}}$) = 29 cells, *N* ($_{\text{Swainsonine}}$) = 19, *N* ($_{\text{GalNAc-}\alpha\text{-}O\text{-benzyl}}$) = 29, *N* ($_{\text{Ac-5SGlcNAc}}$) = 31. Mean Galectin-3 intensity ($_{\text{DMSO}}$) = 2.4±0.1 %, ($_{\text{GalNAc-a-benzyl}}$) = 2.5±0.2 %, ($_{\text{Swainsonine}}$) = 2.4±0.2 %, ($_{\text{Ac-5SGlcNAc}}$) = 0.7±0.1 %. Ordinary one-way ANOVA followed by Tukey-Kramer test. #*P*-value < 0.0001; ##*P*-value < 0.0001; ###*P*-value < 0.0001. Data are means ± SEM.

Figure 5:

e, *N* ($_{\text{Control CRISPR}}$) = 511 cells, *N* ($_{\text{NuMA CRISPR}}$) = 456, *N* ($_{\text{NuMA-wt-GFP}}$) = 377, *N* ($_{\text{NuMA-S1844A-GFP}}$) = 291, *N* ($_{\text{NuMA-S1844T-GFP}}$) = 445. Data are means ± SEM. Mean multipolar cells ($_{\text{Control CRISPR}}$) = 17.04±0.49 %, ($_{\text{NuMA CRISPR}}$) = 74.76±4.43 %, ($_{\text{NuMA-wt-GFP}}$) = 20.15±1.76 %, ($_{\text{NuMA-S1844A-GFP}}$) = 67.97±1.73 %, ($_{\text{NuMA-S1844T-GFP}}$) = 28.75±0.92 %. Ordinary one-way ANOVA followed by Tukey-Kramer test. #*P*-value = 0.032; ##*P*-value < 0.0001. ***g***, *N* ($_{\text{NuMA-wt-GFP}}$) = 15 cells, *N* ($_{\text{NuMA-S1844A-GFP}}$)

= 16, *N* ($_{\text{NuMA-S1844T-GFP}}$) = 12. Data are means ± SEM. Mean multipolar cells ($_{\text{NuMA-wt-GFP}}$) = 0.42±0.04, ($_{\text{NuMA-S1844A-GFP}}$) = 0.09±0.02, ($_{\text{NuMA-S1844T-GFP}}$) = 0.41±0.05. Ordinary one-way ANOVA followed by Tukey-Kramer test. #*P*-value < 0.0001, ##*P*-value < 0.0001.

Figure 6:

e, *N* ($_{\text{Control siRNA}}$) = 43 spindles, *N* ($_{\text{Galectin-3 siRNA\#1}}$) = 43, *N* ($_{\text{Galectin-3 siRNA\#2}}$) = 43. Data are mean ± SEM. Mean volume ($_{\text{Control siRNA}}$) = 19.6±2.1 μm^3, ($_{\text{Galectin-3 siRNA\#1}}$) = 35.2±3.6 and ($_{\text{Galectin-3 siRNA\#2}}$) = 32.5±2.3. T- test analysis, **P*-value =0.0001 and ***P*-value =0.0004.

Figure 7:

b, *T=1 min*, *N* ($_{\text{Control siRNA}}$) = 191 cells, *N (*$_{\text{Galectin-3 siRNA\#1}}$) = 134, *N* ($_{\text{Galectin-3 siRNA\#2}}$) = 136. *M*ean "*no MT"* ($_{\text{Control siRNA}}$) = 8.9±1.5 %, ($_{\text{Galectin-3 siRNA\#1}}$) = 47.0±0.2 %, ($_{\text{Galectin-3 siRNA\#2}}$) = 46.6±6.0 %. Mean "*MT foci"* ($_{\text{Control siRNA}}$) = 91.1±1.5 %, ($_{\text{Galectin-3 siRNA\#1}}$) = 53.0±0.2 %, ($_{\text{Galectin-3 siRNA\#2}}$) = 58.3±5.6 %. *T=5 min*, *N* ($_{\text{Control siRNA}}$) = 76 cells, *N* ($_{\text{Galectin-3 siRNA\#1}}$) = 90, *N* ($_{\text{Galectin-3 siRNA\#2}}$) = 69. Mean "*MT foci"* ($_{\text{Control siRNA}}$) = 19.1±1.7 %, ($_{\text{Galectin-3 siRNA\#1}}$) = 47.6±0.8 %, ($_{\text{Galectin-3 siRNA\#2}}$) = 52.0±16.9 %. Mean "*2 spindles"* ($_{\text{Control siRNA}}$) = 40.3±3.0 %, ($_{\text{Galectin-3 siRNA\#1}}$) = 11.9±0.7 %, ($_{\text{Galectin-3 siRNA\#2}}$) = 6.1±2.0 %. Mean "*>2 spindles"* ($_{\text{Control siRNA}}$) = 40.6±4.5 %, ($_{\text{Galectin-3 siRNA\#1}}$) = 40.5±0.7 %, ($_{\text{Galectin-3 siRNA\#2}}$) = 41.9±16.5 %. *T=20 min*, *N* ($_{\text{Control siRNA}}$) = 273 cells, *N* ($_{\text{Galectin-3 siRNA\#1}}$) = 119, *N* ($_{\text{Galectin-3 siRNA\#2}}$) = 163. Mean "*2 spindles"* ($_{\text{Control siRNA}}$) = 82.6±0.9 %, ($_{\text{Galectin-3 siRNA\#1}}$) = 53.0±3.8 %, ($_{\text{Galectin-3 siRNA\#2}}$) = 50.9±4.6 %. Mean "*>2 spindles"* ($_{\text{Control siRNA}}$) = 17.4±0.9 %, ($_{\text{Galectin-3 siRNA\#1}}$) = 47.0±3.8 %, ($_{\text{Galectin-3 siRNA\#2}}$) = 49.1±4.6 %. Two-way ANOVA followed by Tukey's multiple comparison test. #*P*-value < 0.0001; ##*P*-value < 0.05; ###*P*-value < 0.01.

<u>Supplementary movies 1 and 2:</u>

Live cell imaging of NuMA-GFP localization in spindle pole plane in control and Galectin-3-silenced cells, respectively.

Supplementary Figures

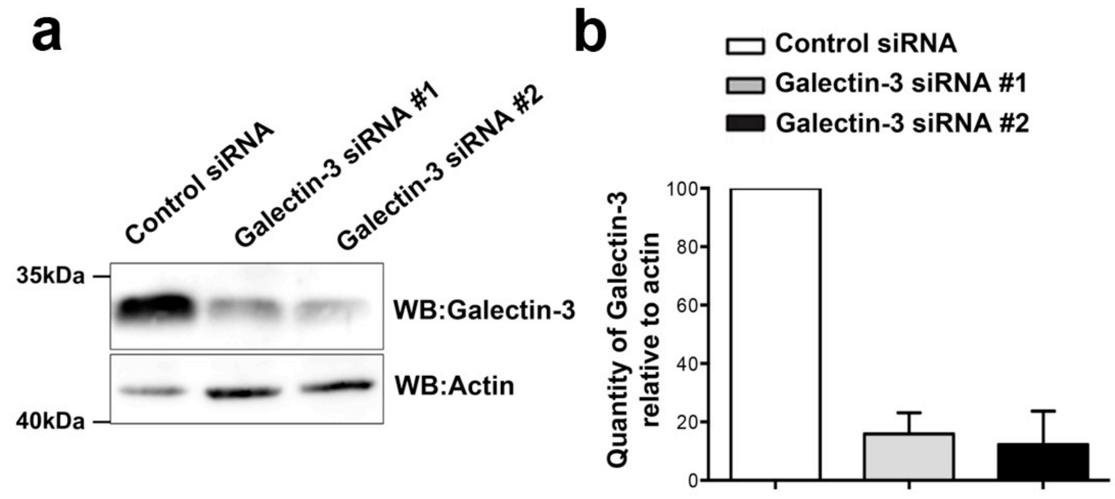

Supplementary Figure 1: Efficiency of Galectin-3 silencing through transient transfections with siRNAs in Hela cells. ***a***, Western blot detection of Galectin-3 and actin amounts in total lysates from Hela cells transfected with control siRNA, Galectin-3 siRNA #1 or Galectin-3 siRNA #2. ***b***, Statistical analysis of Galectin-3 amounts relative to actin amounts in control siRNA (*white*), Galectin-3 siRNA #1 (*grey*) or Galectin-3 siRNA #2 (*black*). The analysis was performed based on three independent experiments. Percentage of Galectin-3 expression in Control siRNA cells = 100 %, in Galectin-3 siRNA#1 cells = 15.95±7.15 %, and in Galectin-3 siRNA#2 = 12.43±6.52 %.

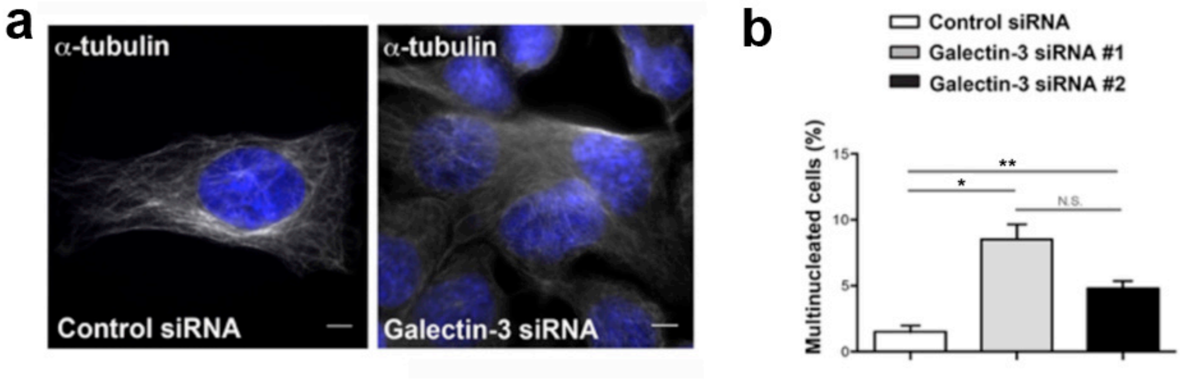

Supplementary Figure 2: Loss of Galectin-3 leads to an increase in the amount of multinucleated cells in Hela cells. ***a***, Confocal microscopy analysis of the microtubule (MT) network in Hela cells in the presence (*Control siRNA*, *left panel*) or in the absence (*Galectin-3 siRNA*, *right panel*) of Galectin-3. Immunostaining was performed with a monoclonal anti-α-tubulin antibody (*white*) and nuclei were detected with Hoechst33342 staining (*blue*). Scale bars, 5 µm. ***b***, Statistical analysis of the number of multinucleated cells in control (*Control siRNA*, *white*) or Galectin-3-deprived (*Galectin-3 siRNA #1*, *grey*, and *Galectin-3 siRNA #2*, *black*) Hela cells. Three independent experiments were carried out. $N_{(\text{Control siRNA})}$ = 1852 cells; $N_{(\text{Galectin-3 siRNA \#1})}$ = 630 cells; $N_{(\text{Galectin-3 siRNA \#2})}$ = 1423 cells. Data are means ± SEM; Mean of multinucleated cells $_{(\text{Control siRNA})}$ = 1.5±0.8 %, mean of multinucleated cells $_{(\text{Galectin-3 siRNA\#1})}$ = 8.5±1.9 %, and mean of multinucleated cells $_{(\text{Galectin-3 siRNA\#2})}$ = 4.8±0.9 %. T-test, * *P*-value = 0.013, ** *P*-value = 0.011.

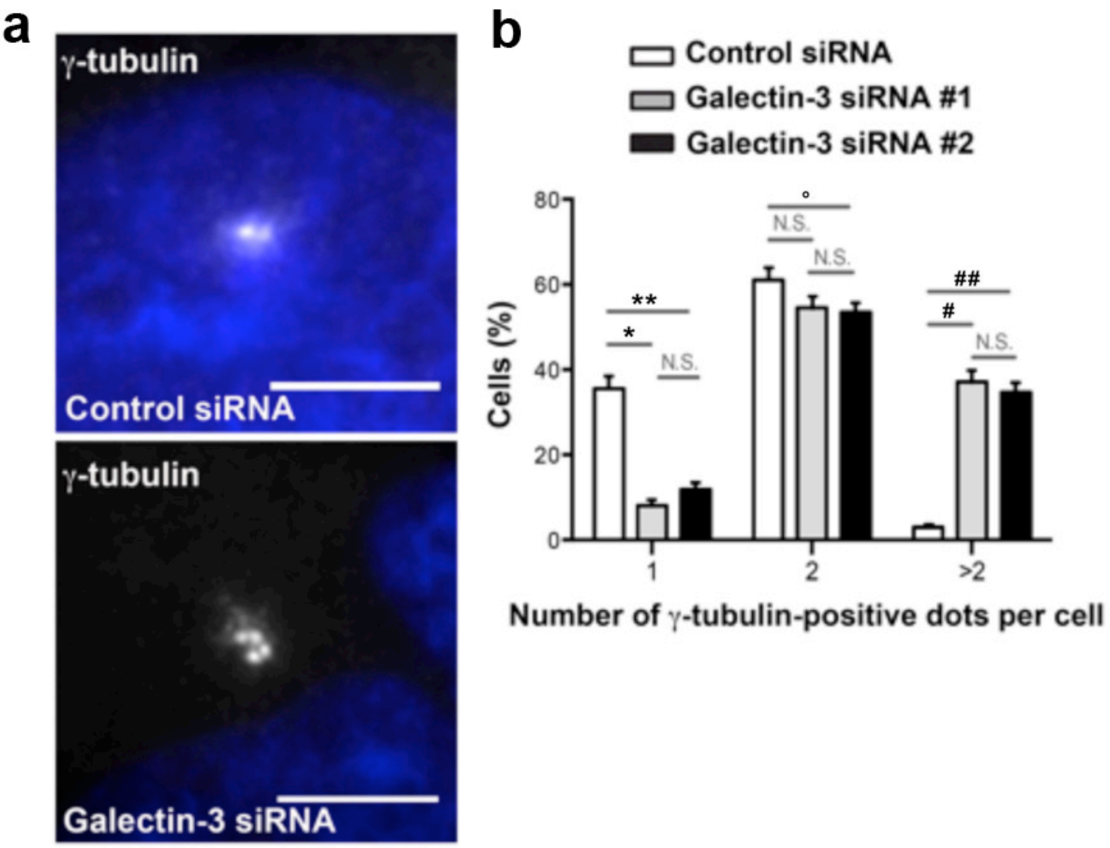

Supplementary Figure 3: Loss of Galectin-3 leads to an increase in the amount of cells with suprenumerous centrioles in Hela cells. ***a***, Confocal microscopy analysis of centrosomes after γ-tubulin immunostaining (*white*) in control (*Control siRNA*) and Galectin-3 silenced (*Galectin-3 siRNA*) Hela cells. Nuclei were detected by Hoechst 33342 staining (*blue*). Scale bars, 5µm. ***b***, Statistical analysis of the number of γ-tubulin-positive dots per cell in control (*white*) and Galectin-3 deprived (*grey* and *black*) Hela cells. Three independent experiments were carried out. n ($_{\text{Control siRNA}}$) = 954 cells; n ($_{\text{Galectin-3 siRNA \#1}}$) = 1077 cells; n ($_{\text{Galectin-3 siRNA \#2}}$) = 895 cells. Data are mean ± SEM; Percentage of Control siRNA cells with 1 γ-tubulin-positive dot = 35.7±2.8 %, percentage of Control siRNA cells with 2 γ-tubulin-positive dots = 61.2±2.9 %, percentage of Control siRNA cells with more than 2 γ-tubulin-positive dots = 3.1±0.6 %, percentage of Galectin-3 siRNA#1 cells with 1 γ-tubulin-positive dot = 8.2±1.3 %, percentage of Galectin-3 siRNA#1 cells with 2 γ-tubulin-positive dots = 54.7±2.7 %, percentage of Galectin-3 siRNA#1 cells with more than 2 γ-tubulin-positive dots = 37.2±2.7 %, percentage of Galectin-3 siRNA#2 cells with 1 γ-tubulin-positive dot = 11.9±1.7 %, percentage of Galectin-3 siRNA#2 cells with 2 γ-tubulin-positive dots = 53.4±2.4 %, percentage of Galectin-3 siRNA#2 cells with more than 2 γ-tubulin-positive dots = 34.7±2.2 %. T-test, * *P*-value = 3.10^{-14}; ** *P*-value = 4.10^{-10}; ° *P*-value = 0.03; # *P*-value = 7.10^{-11}; ## *P*-value = 1.10^{-15}.

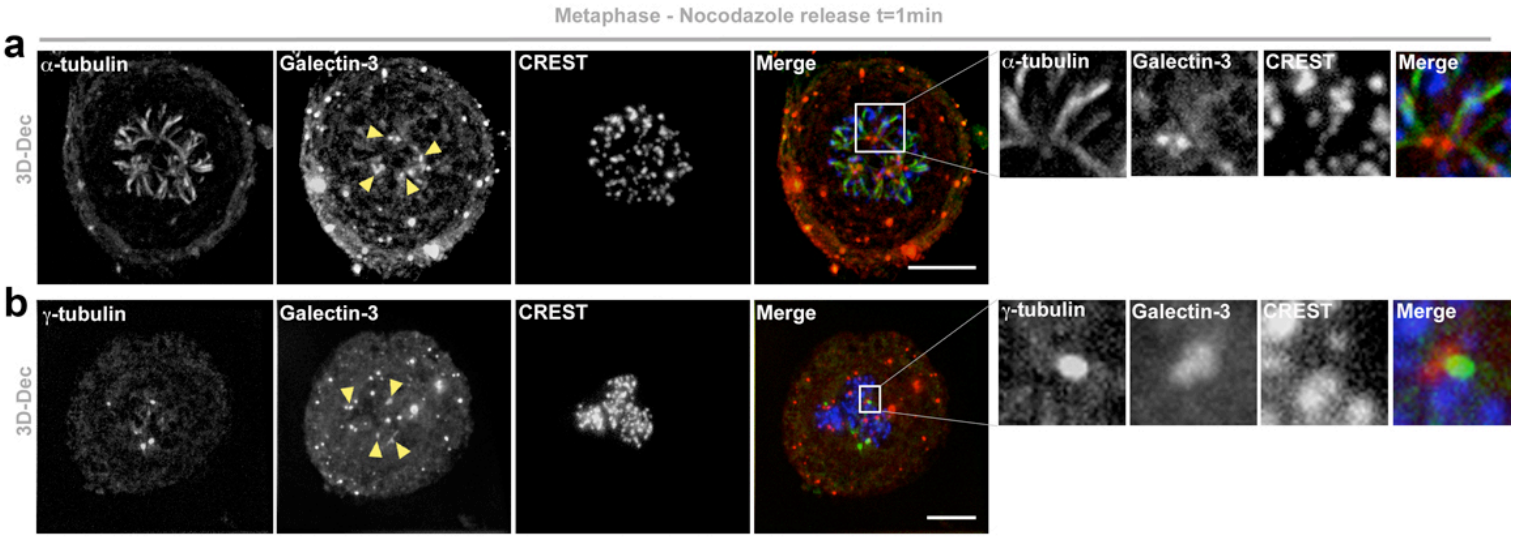

Supplementary Figure 4: Galectin-3 localizes close to MT nucleation complexes after nocodazole treatment. Fluorescence microscopy analysis of Galectin-3 distribution during spindle formation after nocodazole treatment, wash out and 1 min MT regrowth. Hela cells were immunostained with α-tubulin or γ-tubulin (***a*** and ***b*** respectively, *green*), Galectin-3 (*red*) and CREST (*blue*) and 3D-deconvolution has been performed. Galectin-3 is located at early mitotic MT nucleation sites on chromatin (*yellow arroheads*). Closed-ups of MT regrowth foci are presented in inserts on the right panels. Nuclei were detected by Hoechst 33342 staining (*blue*). Scale bars, 5 μm.

	Protein Name	Score	Peptides
(Q5SW79)	Centrosomal Protein of 170 kDa, short name: CEP170	55,08	2
(Q13409)	Dynein, cytoplasmic 1, intermediate chain 2, short name: DYNC1I2	91,63	2
(Q9BW19)	Kinesin-like protein KIFC1, short name: KIFC1, HSET	77,56	2
(Q14980)	Nuclear mitotic apparatus protein 1, short name: NUMA1	74,88	2
(P57740)	Nuclear pore complex protein Nup107, short name: NUP107	60,73	2
(Q8NHV4)	Neural precursor cell expressed developmentally down-regulated protein 1, short name: NEDD1	97,13	4
(O43663)	Protein regulator of cytokinesis 1, short name: PRC1	31,06	2
(Q8TF72)	Protein Shroom3, short name: SHROOM3	39,6	2
(Q15019)	Septin-2, short name: SEPTIN-2	68,13	3
(P04350)	Tubulin beta-4A chain, short name: TBB4A	67,13	4

Supplementary Figure 5: Identification of Galectin-3 ligands in mitotic HeLa cells. By mass spectrometry analyses, a limited number of Galectin-3 co-immunoprecipitated proteins were identified in metaphase enriched HeLa cell lysates and are listed here. Are displayed the Swissprot accession numbers, the protein names, the identification scores and the number of matched peptides.

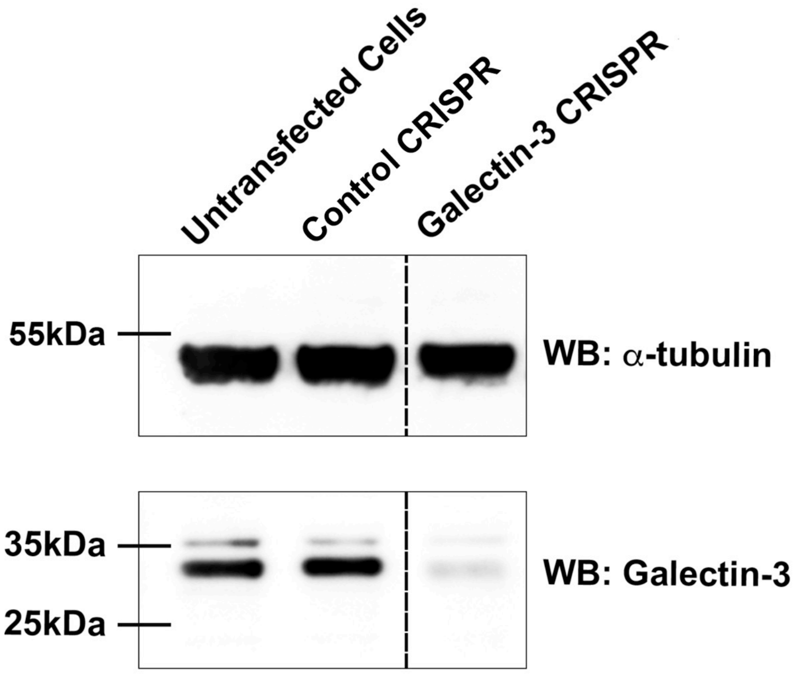

Supplementary Figure 6: Efficiency of Galectin-3 depletion through CRISPR/Cas9 strategy in Hela cells. Western blot detection of Galectin-3 and α-tubulin amounts in total lysates from Hela cells that were transfected with control CRISPR or Galectin-3 CRISPR KO plasmids.

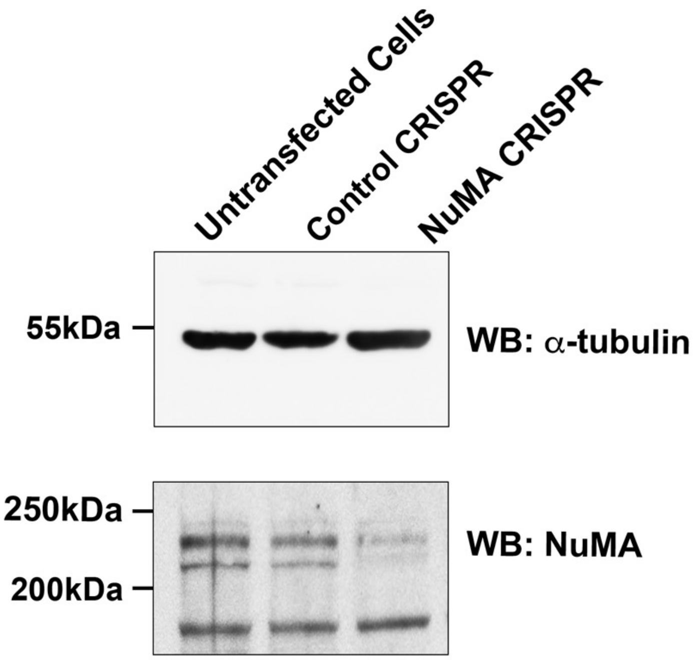

Supplementary Figure 7: Efficiency of NuMA depletion through CRISPR/ Cas9 strategy in Hela cells. Western blot detection of NuMA and α-tubulin amounts in total lysates from Hela cells that were transfected with control CRISPR or NuMA CRISPR KO plasmids.

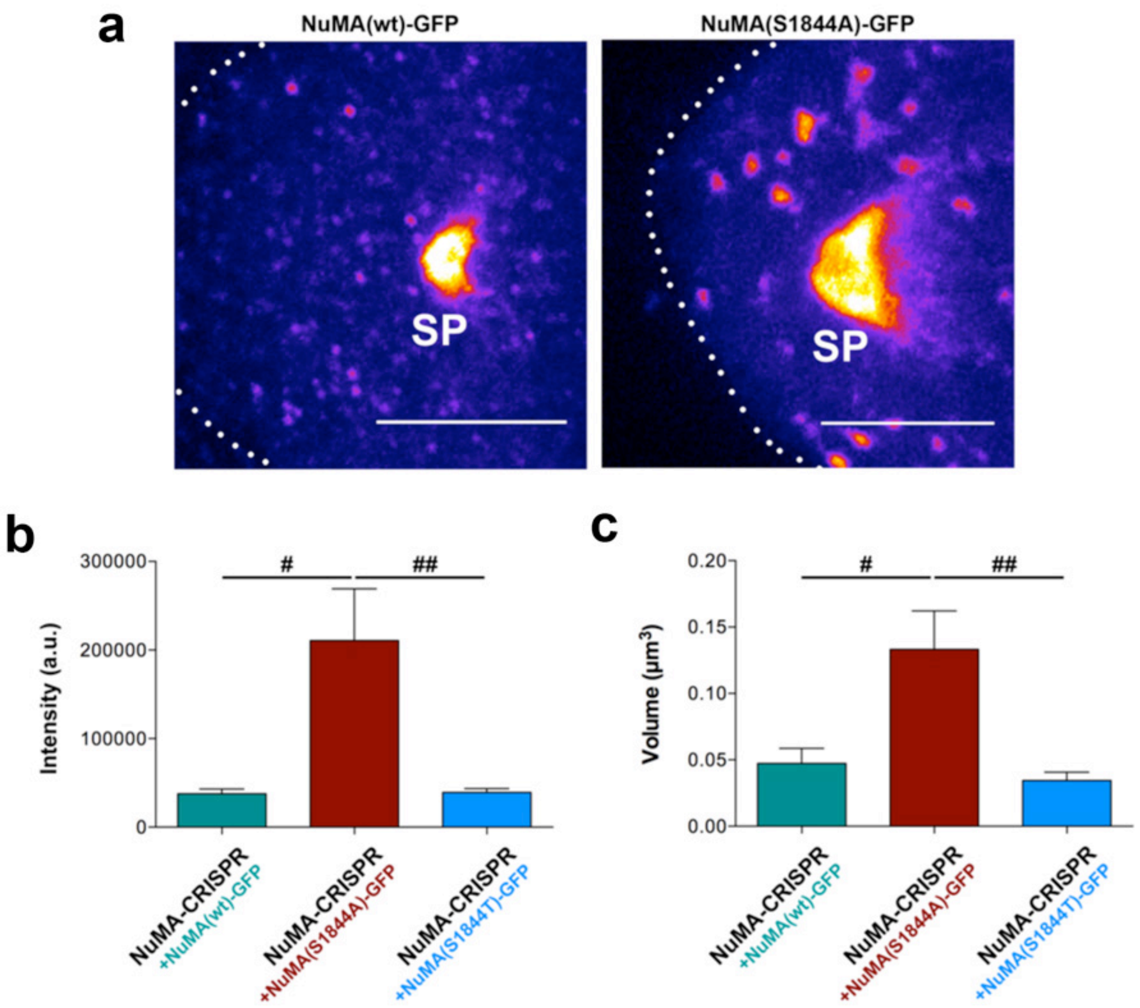

Supplementary Figure 8: Mutation of NuMA *O*-GlcNAc-glycosylation site leads to defects in intensity and volume of cytoplasmic foci. ***a***, Confocal microscopy and max projection analysis of GFP-tagged NuMA(wt) (*left panel*) and NuMA(S1844A) (*right panel*) at spindle pole of bipolar metaphase. SP, spindle pole. Cell plasma membrane is demarcated with a white dotted line. Scale bars, 10μm. ***b***, quantification of the intensity of NuMA foci in the area of the cell cortex in NuMA(wt)-GFP, NuMA(S1844A)-GFP or NuMA(S1844T)-GFP cells. N $(_{\text{NuMA(wt)-GFP}})$ = 9 cells , N $(_{\text{NuMA(S1844A)-GFP}})$ = 10 cells, and N $(_{\text{NuMA(S1844T)-GFP}})$ = 10 cells. Data are means ± SEM. Mean GFP foci intensity $(_{\text{NuMA(wt)-GFP}})$ = 37620±5448, mean GFP foci intensity $(_{\text{NuMA(S1844A)-GFP}})$ = 210786±58372 and mean GFP foci intensity $(_{\text{NuMA(S1844T)-GFP}})$ = 39372±4319. Ordinary One-way ANOVA followed by tukey's multiple comparisons test; # *P*-value = 0.0053 and ## *P*-value = 0.0046. ***c***, quantification of the volume of NuMA foci in the area of the cell cortex in NuMA(wt)-GFP, NuMA(S1844A)-GFP or NuMA(S1844T)-GFP cells. N $(_{\text{NuMA(wt)-GFP}})$ = 9 cells , N $(_{\text{NuMA(S1844A)-GFP}})$ = 10 cells, and N $(_{\text{NuMA(S1844T)-GFP}})$ = 10 cells. Data are means ± SEM. Mean GFP foci volume $(_{\text{NuMA(wt)-GFP}})$ = 0.047±0.011 μm^3, mean GFP foci volume $(_{\text{NuMA(S1844A)-GFP}})$ = 0.133±0.029 μm^3 and mean GFP foci volume $(_{\text{NuMA(S1844T)-GFP}})$ = 0.035±0.006 μm^3. Ordinary One-way ANOVA followed by tukey's multiple comparisons test; # *P*-value = 0.0095 and ## *P*-value = 0.0022.

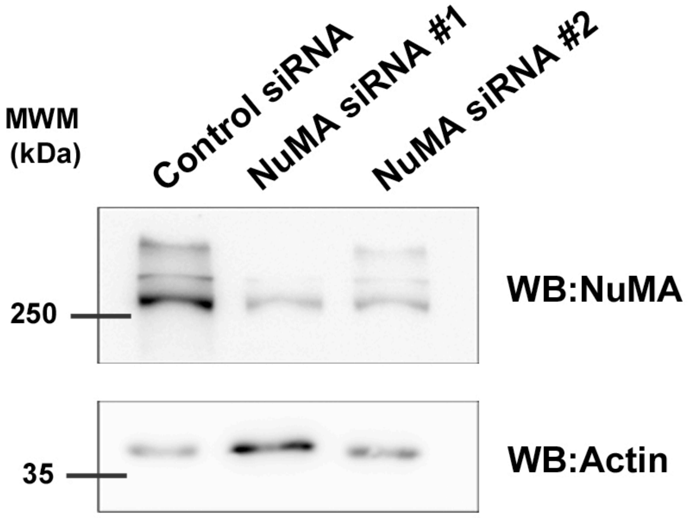

Supplementary Figure 9: Efficiency of NuMA silencing through transient siRNA transfections in Hela cells. Western blot detection of NuMA and actin amounts in total lysates from Hela cells that were transfected with control siRNA, NuMA siRNA #1 or NuMA siRNA #2.

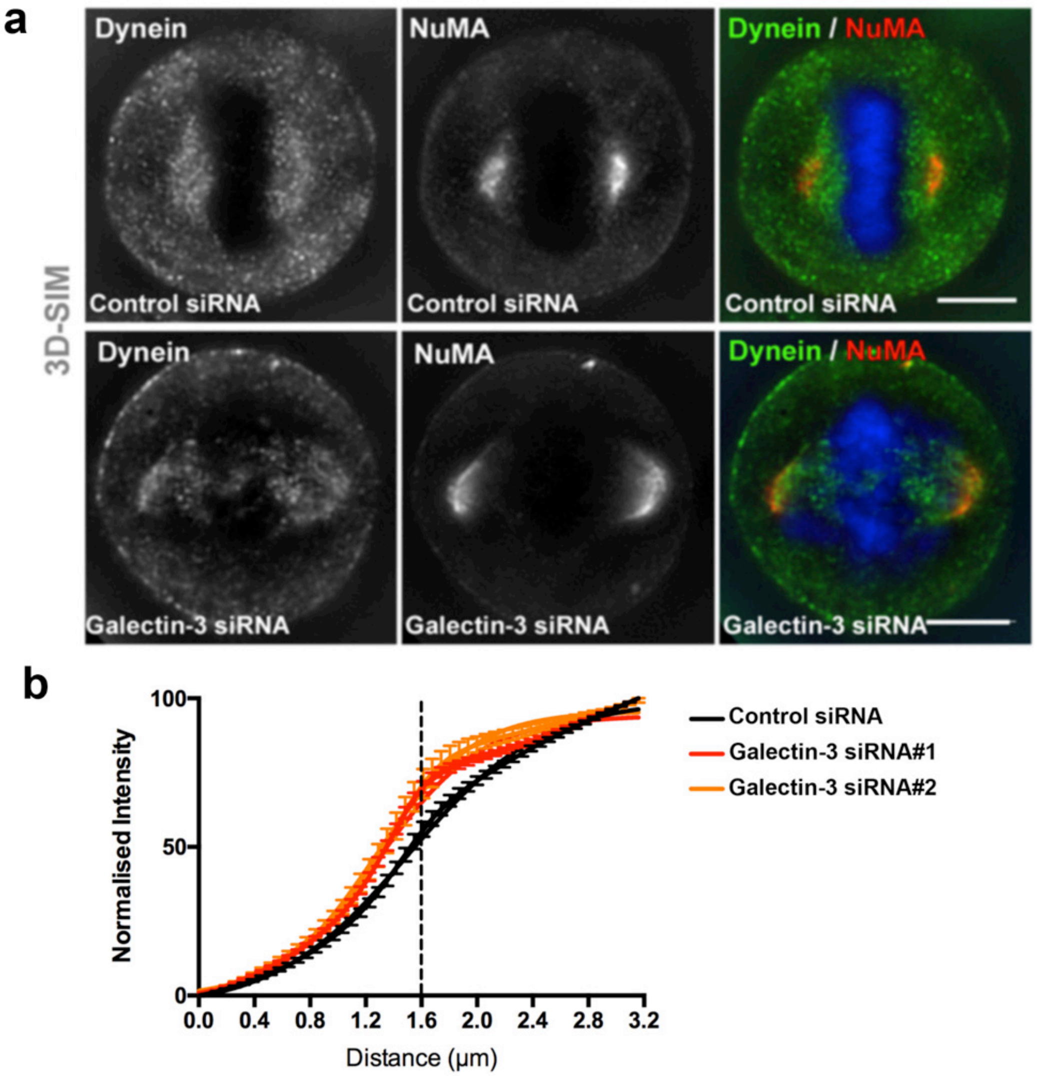

Supplementary Figure 10: Loss of Galectin-3 leads to accumulation of NuMA and dynein at the metaphase cell cortex in Hela cells. *a,* 3D-SIM microscopy analysis of dynein (*green*) and NuMA (*red*) in control (*Control siRNA*, *upper panel*) and Galectin-3 silenced (*Galectin-3 siRNA*, *lower panel*) metaphase Hela cells. Scale bars, 5 µm. ***b,*** quantification of NuMA intensity at the cell cortex in control and Galectin-3 siRNA cells. Dashed line corresponds to the position of the membrane. N ($_{\text{Control siRNA}}$) = 19 cells , N ($_{\text{Galectin-3 siRNA\#1}}$) = 16 cells, and N ($_{\text{Galectin-3 siRNA\#2}}$) = 10 cells. Data are means ± SEM.

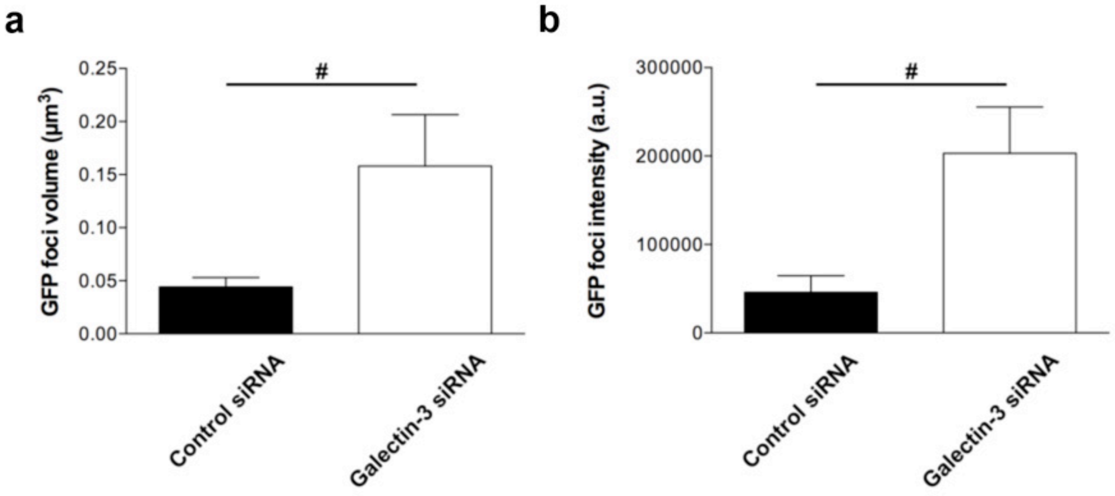

Supplementary Figure 11: Galectin-3 depletion leads to defects in NuMA localization. ***a***, quantification of the volume of NuMA foci around the cell cortex in Control siRNA or Galectin-3 siRNA cells. N ($_{\text{Control siRNA}}$) = 3 cells and N ($_{\text{Galectin-3 siRNA}}$) = 4 cells. Data are means ± SEM. Mean GFP foci volume ($_{\text{Control siRNA}}$) = 0.044±0.005 μm^3 and mean GFP foci volume ($_{\text{Galectin-3 siRNA}}$) = 0.158±0.024 μm^3. T-test; # *P*-value = 0.011 ***b***, quantification of the intensity of NuMA foci around the cell cortex in Control siRNA or Galectin-3 siRNA cells. N ($_{\text{Control siRNA}}$) = 3 cells and N ($_{\text{Galectin-3 siRNA}}$) = 4 cells. Data are means ± SEM. Mean GFP foci intensity ($_{\text{Control siRNA}}$) = 45872±10827 and mean GFP foci intensity ($_{\text{Galectin-3 siRNA}}$) = 203130±26201. T-test; # *P*-value = 0.0046.
